# An application of machine learning to haematological diagnosis

**DOI:** 10.1038/s41598-017-18564-8

**Published:** 2018-01-11

**Authors:** Gregor Gunčar, Matjaž Kukar, Mateja Notar, Miran Brvar, Peter Černelč, Manca Notar, Marko Notar

**Affiliations:** 1 Smart Blood Analytics Swiss SA, CH-7000 CHUR, Switzerland; 2 Centre for Clinical Toxicology and Pharmacology, Division of Internal Medicine, University Medical Centre Ljubljana, SI-1000 Ljubljana, Slovenia; 3 Department of Haematology, Division of Internal Medicine, University Medical Centre Ljubljana, SI-1000 Ljubljana, Slovenia

## Abstract

Quick and accurate medical diagnoses are crucial for the successful treatment of diseases. Using machine learning algorithms and based on laboratory blood test results, we have built two models to predict a haematologic disease. One predictive model used all the available blood test parameters and the other used only a reduced set that is usually measured upon patient admittance. Both models produced good results, obtaining prediction accuracies of 0.88 and 0.86 when considering the list of five most likely diseases and 0.59 and 0.57 when considering only the most likely disease. The models did not differ significantly, which indicates that a reduced set of parameters can represent a relevant “fingerprint” of a disease. This knowledge expands the model’s utility for use by general practitioners and indicates that blood test results contain more information than physicians generally recognize. A clinical test showed that the accuracy of our predictive models was on par with that of haematology specialists. Our study is the first to show that a machine learning predictive model based on blood tests alone can be successfully applied to predict haematologic diseases. This result and could open up unprecedented possibilities for medical diagnosis.

## Introduction

Machine learning has undergone significant development over the past decade and is being used successfully in many intelligent applications covering a wide array of data related problems^1^. One of the most intriguing questions is whether machine learning can be successfully applied to the field of medical diagnostics. Moreover, there is a question as to what kind of data are needed. Several examples of successful applications of machine learning methods in specialized medical fields exsist^2–6^. Recently, a model capable of classifying skin cancers based on images of the skin was presented that achieves a level of competence comparable to that of a dermatologist^7^. There are however, no successful applications of machine learning that tackle broader and more complex fields in medical diagnosis, such as haematology.

Medical diagnosis is the process of determining which disease best explains a person’s symptoms and signs. For a physician to determine a differential diagnosis and make quick treatment plans, medical knowledge, skills and experience all play a significant role^8^. In a diagnostic procedure, the available information is complemented by additional data gathering, which can be obtained from a patient’s medical history, a physical examination and from various diagnostic tests, including clinical laboratory tests. Laboratory tests are used to confirm, exclude, classify or monitor diseases and to guide treatment^9^. However, the true power of laboratory test results is frequently underestimated because clinical laboratories tend to report test results as individual numerical or categorical values, and physicians concentrate mainly on those values that fall outside a given reference range^10^.

The clinical diagnosis of haematological diseases is primarily based on laboratory blood tests—and even the most skilled haematology specialist can overlook patterns, deviations and relations between the increasing numbers of blood parameters that modern laboratories measure. In contrast, machine learning algorithms can easily handle hundreds of attributes (parameters), and they are capable of detecting and utilizing the interactions among these numerous attributes, which makes this field of medicine particularly interesting for machine learning applications. Our hypothesis was that the “fingerprint” of a certain haematological disease found in the values of blood test results would be sufficient for a machine learning based predictive model to suggest a plausible diagnosis, provided that the machine were trained on a sufficiently large dataset of medical cases that include clinical laboratory blood tests and that were labelled with a correct diagnosis determined by a haematology specialist who has utilized all of the diagnostic procedures necessary to confirm it.

In this study, we describe and evaluate two Smart Blood Analytics (SBA) haematological-predictive models based on two different sets of clinical laboratory blood test results (with different numbers of blood parameters), coded diseases and their evaluations. We evaluated both models using stratified ten-fold cross-validation of 8,233 cases, as well as 20 additional randomly selected haematological cases, and compared their performance against an evaluation performed by haematological and internal medicine specialists. We also present an illustrative example showing how our SBA predictive model can help improve the speed of the diagnostic process.

## Materials and Methods

### Study setting and population

We collected data provided by the Clinical Department of Haematology for patients admitted to the University Medical Centre of Ljubljana (UMCL) between 2005 and 2015. The hospital is a tertiary referral centre located in Ljubljana, Slovenia and serves a local population of 400,000 inhabitants. For each admitted adult patient (18 + years of age), we collected anonymized laboratory blood test results and their diagnoses made on admission and discharge. To minimize any bias from previous treatments we considered only a patient’s first admittance during the sampling period. In total, we collected data on 8,233 cases for which 371,341 laboratory blood tests were performed. We then manually curated the data and identified 181 attributes, consisting of 179 different blood tests that were performed at least 10 times (see Supplementary Table S1). We also included the genders and ages of the patients. On average, 24.9% (45 parameters) were measured in every case.

From these 181 attributes, we further selected a reduced subset that contained only 61 parameters (see Supplementary Table S1). The 50 most frequently measured basic parameters, including gender and age, as well as 11 frequently analysed haematological parameters (suggested by expert physicians) were selected. This set comprises the most frequently measured parameters for the haematological patients and was chosen for practical reasons. This set excludes rarely measured parameters that are used to confirm specific diagnoses. Parameter selection was therefore performed according to frequency of use rather than on estimated importance. On average, 66.4% (41 parameters) were measured for every case.

To compare the diagnostic performance of physician with those of our predictive models, we selected an additional 20 random anonymous cases who received their first haematological diagnoses in 2016 or 2017 (see Supplementary Data S2). The data included laboratory blood tests, gender and age from 20 adult patients: 10 female and 10 male.

For recording purposes, the UMCL uses a modified International Statistical Classification of Diseases and Related Health Problems (ICD). Our learning models used recorded diseases to three-characters deep in the ICD hierarchy. In total, 43 different haematological categories of diseases were identified among the 8,233 cases analysed (Table 1, Supplementary Table S3).

**Table 1:** Top ten most prevalent categories of diseases in UMCL, Division of Haematology.

ICD code	Disease Category	Frequency	Prevalence
D47	Other neoplasms of uncertain or unknown behaviour of lymphoid, haematopoietic and related tissue	1522	18.5%
D50	Iron deficiency anaemia	1190	14.5%
D69	Purpura and other haemorrhagic conditions	743	9.0%
C90	Multiple myeloma and malignant plasma cell neoplasms	739	9.0%
C91	Lymphoid leukaemia	696	8.5%
C92	Myeloid leukaemia	578	7.0%
D75	Other diseases of blood and blood-forming organs	547	6.6%
D46	Myelodysplastic syndromes	457	5.6%
D64	Other anaemias	218	2.7%
D45	Polycythaemia vera	204	2.5%
Other		1339	16.1%

### Utilized machine learning algorithms

#### Support Vector Machine (SVM)

The basic idea of the SVM^11^ is to place an optimal class-separating hyperplane in the space of (usually transformed) original attributes. If the learning examples are linearly separable in the transformed space, then there are generally several possible separating hyperplanes. The optimal hyperplane is equally (and therefore most) distant from the nearest examples (*support vectors*) from different classes. The optimal hyperplane is therefore selected to maximize the margin (the distance between the hyperplane and its support vectors). The transformation of the original attribute space is characterized by the use of a corresponding *kernel* function. Among the available kernel functions, linear and radial basis functions are the most popular. In our experiments we used the scikit-learn implementation SVC, which is based on the libsvm library^12^. With respect to the tunable parameters, we experimented with both linear and radial basis kernels. The Γ parameter was calculated by the heuristic *1/number of attributes*. The penalty parameter *C* was tuned using internal cross-validation in the training set. We found that using values higher than the default value of 1 improved the classification accuracy—but only by approximately 2%. The results reported in Table 2 were obtained with C = 100 but were still nowhere near the results of random forests.

**Table 2:** Comparison of different machine learning methods. Average accuracy and standard deviation from 10-fold stratified cross-validation on full data (181 parameters). The SVMs used scaled parameter values; other methods used original values.

Method	Naïve Bayes	Decision Tree	SVM (linear)	SVM (RBF)	Random forest
Predictive accuracy	0.07 ± 0.006	0.42 ± 0.01	0.52 ± 0.01 (scaled)	0.54 ± 0.01 (scaled)	0.59 ± 0.005

#### Naïve Bayesian classifier

The task of the Bayesian learning algorithm^13^ is to use learning examples to estimate conditional class probabilities *P*(*class|*{*attribute* = *value*}) for all attribute subsets as well as unconditional probabilities *P*(*class*). In practice, however, calculating all possible conditional probabilities is both infeasible (due to the combinatorial explosion problem) and usually impossible (due to lack of data); therefore, the naive Bayesian classifier assumes the conditional independence of attributes with respect to the class. Under this assumption, it is sufficient to calculate only the first-order conditional probabilities *P*(*class|attribute* = *value*). Our experiments used the scikit-learn implementation, GaussianNB. This implementation has only one tunable parameter, class priors, however because that value is estimated from the training data, we performed no further tuning.

#### Random forest

One of the most powerful approaches in machine learning are ensemble methods. An ensemble uses multiple learning algorithms to obtain a final predictive performance that is often better than the performance obtainable from any of the constituent learning algorithms alone. The *random forest* algorithm^14^ is a special type of ensemble method. Ensemble methods^15,16^ combine *weak learners* to form a *strong learner*. A random forest consists of many small decision or regression trees. Each tree, individually, is a weak learner; however, all the trees (i.e., a forest) taken together form a strong learner.

Several authors have shown that random forest algorithms perform well in most problem domains^17^, including medical diagnostics^18,19^. When compared with hundreds of other machine learning approaches applied to many datasets^20^, random forests have emerged as the best performer overall.

Random forests are very fast both for training and for prediction because they can be efficiently parallelized. They perform very well without any parameter tuning, however, they may overfit particularly noisy data. Random forests are also able to address unbalanced data, missing data and data with huge numbers of attributes and classes (for classification purposes). While our data has low noise (all the blood parameters are determined automatically), it is highly dimensional (181 attributes/parameters) and there are many missing values (on average, < 25% of the parameters were measured for each patient) and a relatively high number of classes (43), making random forests the logical choice. Our early experiments exploring other popular machine learning methods (i.e., decision trees, SVM and Naive Bayesian classifiers) resulted in significantly lower performance scores (Table 2).

**Figure 1:**
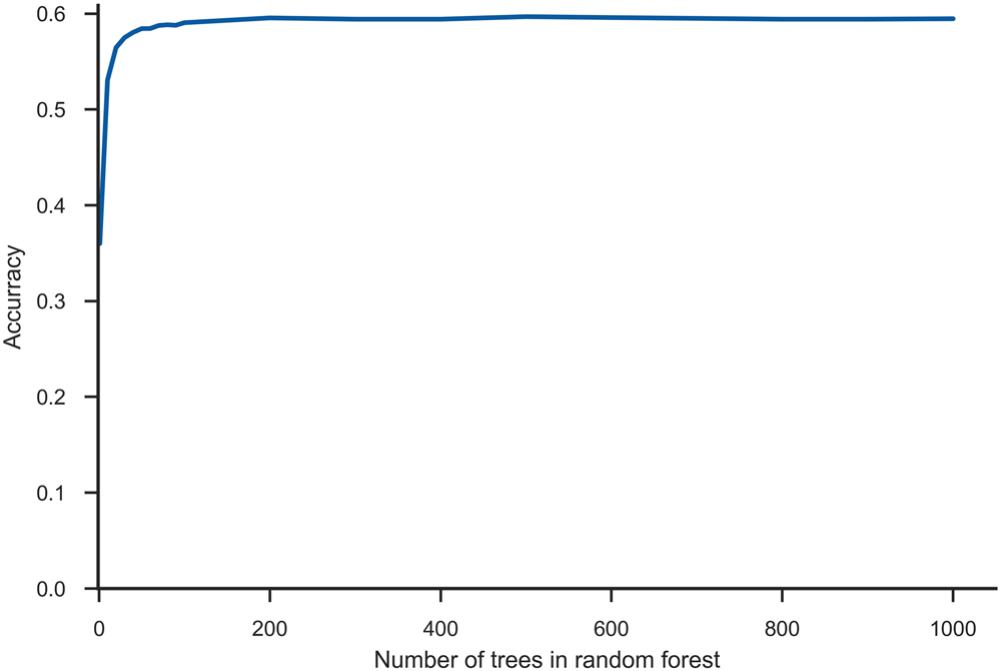
Number of decision trees in random forest. The maximum accuracy is achieved at 200 trees. Further increasing the number of trees increases the training and execution time without significant accuracy benefits, however it slightly reduces the variance.

We also experimented with the number of decision trees used in the random forest and achieved optimal accuracy from 200 trees onward (Fig. 1). However, to reduce the variance (see Table 2), random forests of 500 trees were used in our models.

### Smart Blood Analytics algorithm

The machine learning pipeline (the SBA algorithm) consists of several processing stages:

1. Data acquisition: acquiring raw data from the database.
2. Data filtering: selecting only those blood tests performed at the start of the treatment and at final diagnosis as the machine learning subset.
3. Data preprocessing: canonizing blood parameters (matching them with our reference parameter database, filtering out erroneous values (outliers) and handling missing values (imputation).
4. Data modelling: building the predictive model.
5. Evaluation: evaluating the predictive model with stratified 10-fold cross-validation).

When acquired, most blood parameters were already in SI units; those that were not were recalculated. These values were used for training without any scaling and corrections. We also experimented with scaling all parameters to the interval [−1, 1] centred around each parameter’s median. However, with the exception of SVM, no significant differences were observed compared to using original values. Therefore, because using the original values as parameter values is more comprehensible (reference interval), the original values were used in the final experiments with random forests. No further feature extraction techniques (such as PCA) were used.

**Figure 2:**
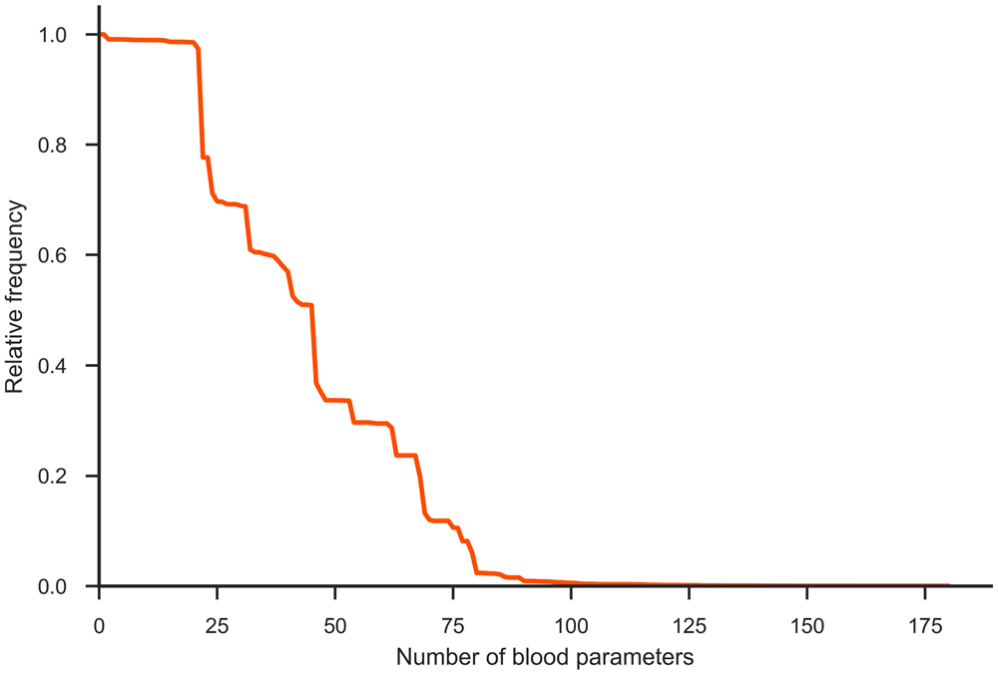
Missing data. Blood parameters ordered by decreasing relative frequency. Most parameter values 75.1% are unknown (missing).

With respect to missing values (75.1%), the available haematological data are rather specific. This is because different blood tests are performed for different suspected diseases. Because most machine learning algorithms (especially matrix-based approaches) require a full data matrix, a data imputation step was necessary. We used two approaches. First, we utilized a simple median imputation. First, for each feature (blood parameter), a median value was calculated. Then, for each patient, missing parameter values were replaced by that parameter’s median value. We also experimented with multivariate imputation by chained equation (MICE)^21^; however that approach did not improve the results—probably because of data sparseness (Fig. 2). In total, 75.1% of the values were missing, and for 2/3 of the parameters, more than 80% of the values were missing). For new, previously unseen cases, missing values were replaced with the corresponding parameter median values calculated from the training set.

**Figure 3:**
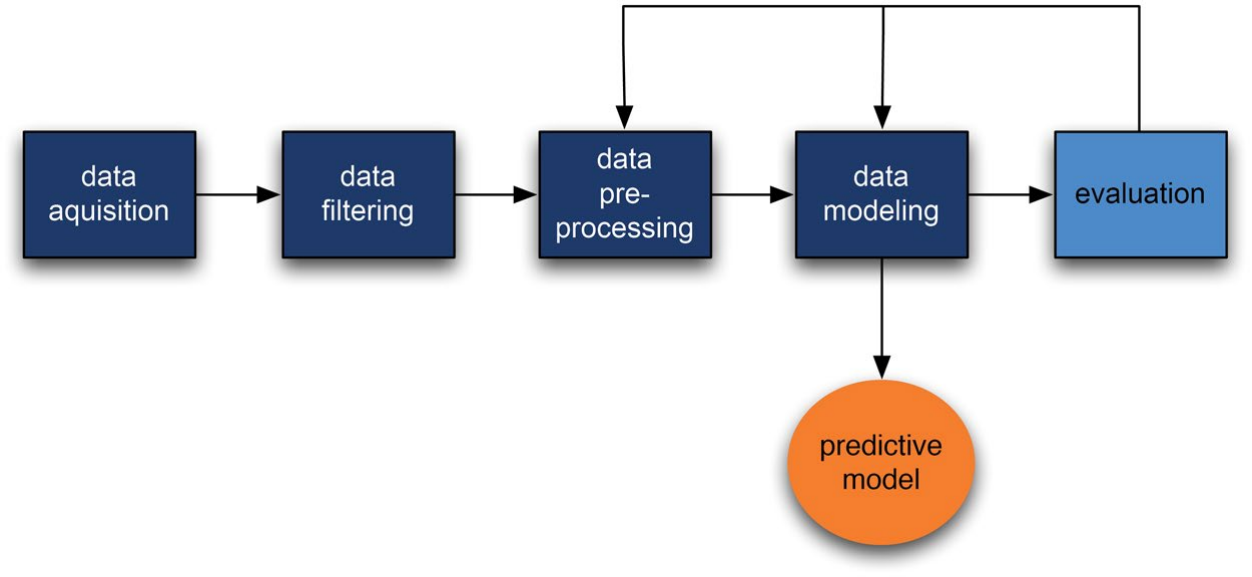
Schematic representation of the Smart Blood Analytics (SBA) algorithm process.

After successful evaluation of the predictive model results, the model was deployed through the Smart Blood Analytics website (www.smartbloodanalytics.com). Otherwise, the process resumed at one of the earlier stages (either data preprocessing or modelling) (Fig. 3).

### Evaluation of predictive models

The models were automatically evaluated using stratified tenfold cross-validation. The folds were selected so that the distribution of diseases was approximately equal in all of the folds. The process was repeated 10 times; for each repetition, one fold was set aside for testing and the remaining 9 folds were used for training. The results were used to perform a statistical comparison of the model’s performance on all 8,233 cases using the Wilcoxon signed-rank test, aggregated into confusion matrices, and performance measures such as diagnostic accuracy, specificity and sensitivity and ROC curves were calculated.

A confusion (or error) matrix is a specific table layout that allows easy visualization of the performance of a supervised machine learning algorithm. Each row of the matrix represents the cases (examples) in a predicted disease (class), while each column represents the cases (examples) in an actual disease (class). Confusion matrices are used both computationally, to derive various performance measures, and visually, to determine which diseases (classes) the algorithm confuses.

Initially, we considered several different machine learning methods: naïve Bayesian classifier, CART decision trees, SVMs with linear and radial basis function kernels and random forests. The results of 10-fold stratified cross-validation are shown in Table 2. Due to its clear superiority, the random forests method was used in subsequent work.

### Evaluating parameter importance

**Figure 4:**
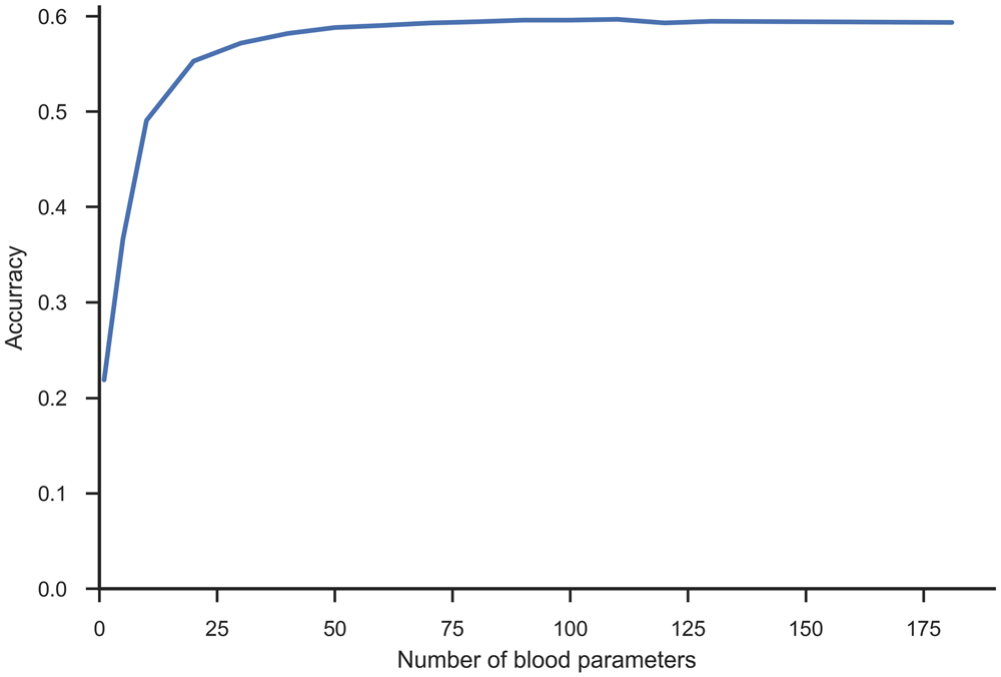
Learning curve. Learning curve with increasing numbers of parameters, ordered by their importance according to ReliefF estimate.

We estimated the importance of all blood parameters with the ReliefF algorithm^22,23^ and ordered them in decreasing fashion. By incrementally including an increasing number of parameters into the models, we obtained the learning curve shown in Fig. 4. The curve increases up to approximately 75 parameters, after which adding additional parameters does not improve the classification accuracy. However, due to the specifics of parameter acquisitions (in larger groups or panels), it serves no purpose to omit some parameters from the same panel. Therefore, in addition to the full parameter set, we also considered the 50 parameters that are most frequently measured (1–50 in Fig. 2), as well as 11 frequently measured haematological parameters suggested by expert physicians.

**Figure 5:**
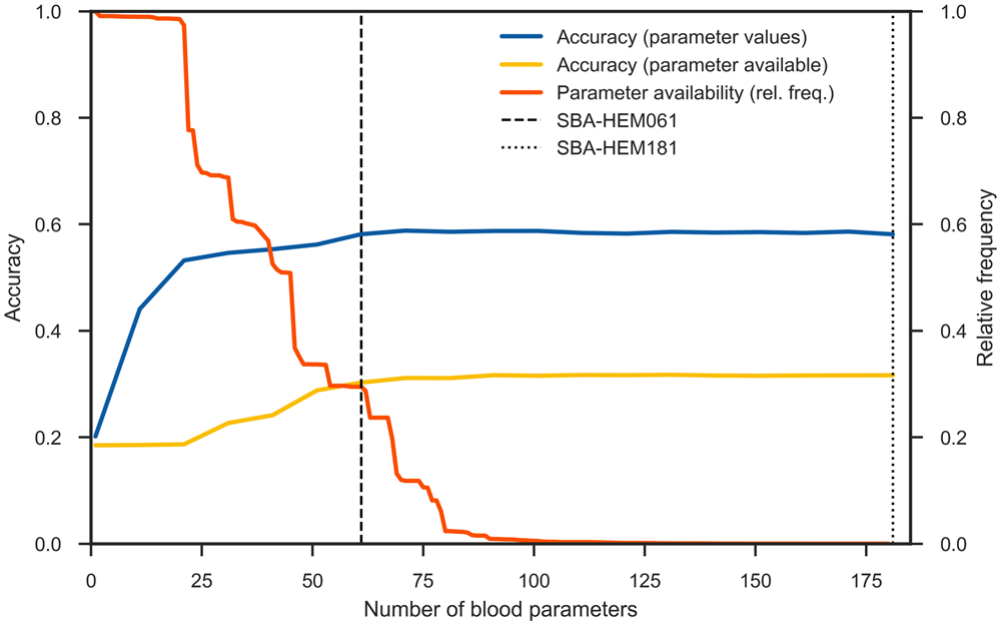
Influences of parameter frequency, presence and actual measured values on model accuracy. The actual parameter value (blue line) and its presence (whether it was measured or not (in yellow line) are depicted, as well as relative frequency of the parameters (the ratio of how many times they were measured to the total number of measurements in orange line). To obtain the blue accuracy curve, the actual parameter values were used for training and testing, while for the yellow accuracy curve, the parameter values were replaced with either 0 (not measured) and 1 (measured), and no imputation was used. The flattening of both accuracy curves indicates that, when the frequently measured parameters are present, the rarely measured parameters contribute little to model accuracy.

We also explored the relationships between parameter frequency and their influence on model accuracy (Fig. 5) by considering both the actual parameter value (depicted in blue) and its availability (whether it was measured, depicted in green), were considered. From our experiments it seems that rarely measured parameters (the long tail in Fig. 5) that are considered as markers for specific diseases contribute very little to model accuracy. This result may be the consequence of data imputation; however, it is likely that the information such parameters carry can be extracted from other, more frequently measured parameters.

### ROC curves

A common approach when evaluating machine learning results for classification (diagnostic) problems is to observe the classification accuracy (true positive rate) obtained by trained classifiers using relevant data sets. However, this approach is valid only under the assumption of uniform error costs (that all errors are equally costly), while this is often not the case in practice. For example, in medical diagnostics, a false positive may result in unnecessary health care costs, while a false negative may endanger the patient’s life due to delayed treatment. It is therefore often better to observe how the trained classifier behaves in a more general setting.

A popular method for visualizing classifier behaviour is to utilize ROC curves^24^^.^ A ROC curve depicts the relation between the classifier’s true positive rate (*sensitivity*) and its false positive rate (1–*specificity*). For two-class problems and *scoring* classifiers (i.e., a classifier that produces a score for each possible class and predicts the class with the maximum score), ROC curves are produced in a straightforward manner by ordering the maximum scores and varying the decision threshold^25^. The one-vs-all approach yields *N* ROC curves that can be useful in observing classifier performance for each class. Multi-class problems with *N* classes can be transformed into *N* two-class problems, in which each problem involves discriminating one class vs all the other *N*–1 classes. The predictive model’s performance is deemed useful for a certain disease (class) when the entire ROC curve is above the diagonal (and as close to the upper left corner as possible). ROC curves near the diagonal are of little use because the predictive model’s performance in such cases is only marginally better than chance.

We applied two approaches suitable for observing overall classifier performance in multi-class problems: *macro-averaging* and *micro-averaging*^26^. In the *macro-averaging* approach, the ROC curves for all *N* diseases were averaged regardless of their frequencies. In the *micro-averaging* approach, the ROC curve was calculated anew, based upon the true positive and false positive rates for all diseases (or equivalently, by weighting ROC curves by the relative frequencies of the respective diseases and then averaging them).

### Clinical test setting

We also performed two clinical tests, first with six haematology specialists and second with eight non-haematology internal medicine specialists with at least eight years of experience. Each individual physician in both groups received the laboratory blood test results from 20 cases. The physicians were asked to determine a maximum of five potential haematological diseases and to sort them in decreasing order of likelihood. The same 20 cases were then used to make predictions with both predictive models.

### Web-based application and graphical representation of predictive model results

**Figure 6:**
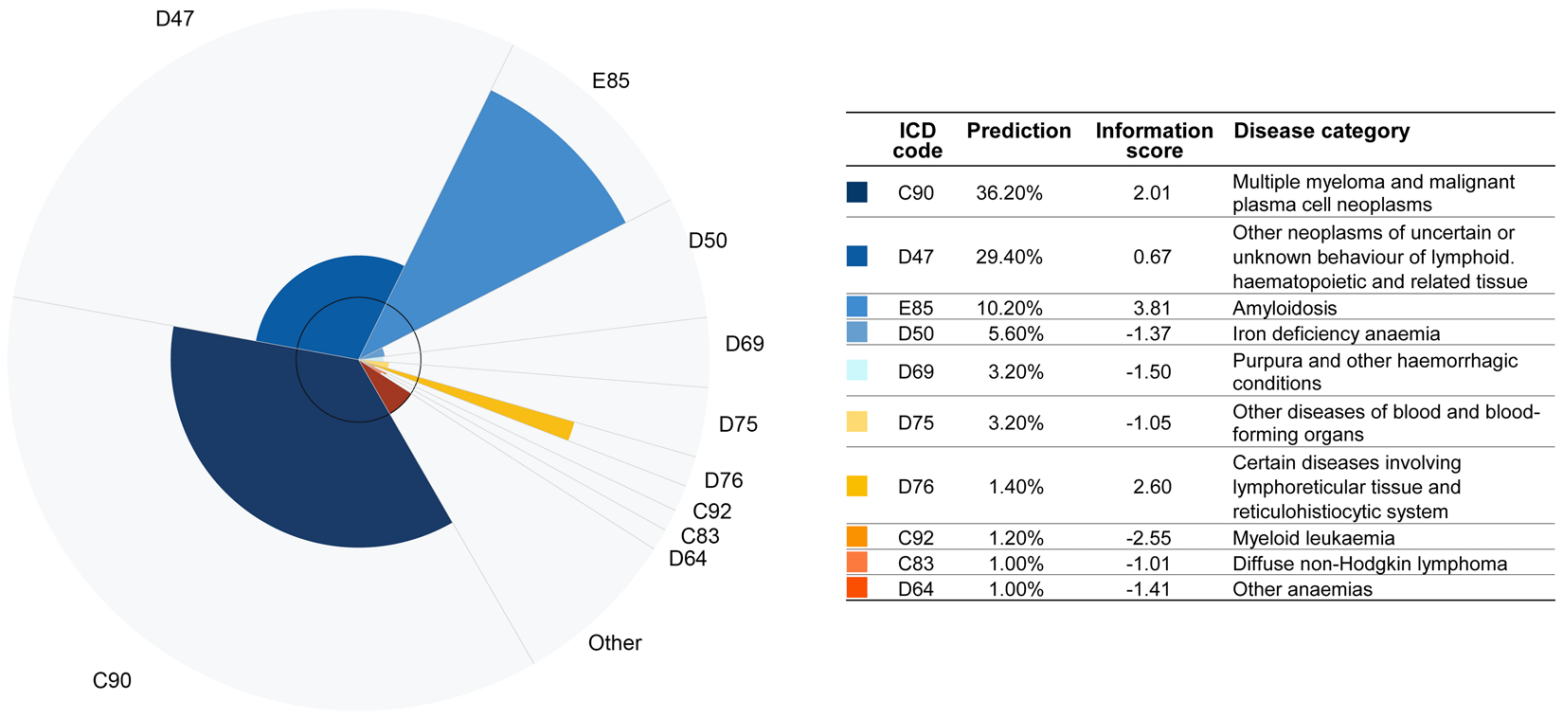
Graphical representation of the predictive model results. The ten most likely diseases are depicted in a polar chart with varying radii. Each chart slice represents a disease whose angle corresponds to the predicted (posttest) disease probability and whose radius is proportional to the logarithm of the ratio between pre- and post-test, the (prevalence) probability or information score.

As part of our work we also developed a web-based application that enables the easy input of blood parameters and produces an innovative representation of the results. This application is available to medical professionals upon registration at www.smartbloodanalytics.com. The application also includes a novel approach for visualizing the machine learning model’s results (Fig. 6) by showing the 10 most likely diseases depicted as a polar chart with varying radii. Each chart segment represents a possible disease. Each segment’s angle corresponds to the predicted (posttest) disease probability. The radius of each segment is proportional to the logarithm of the ratio between the posttest and pretest (prevalence) probability, also called the *information score*. The radius therefore depicts the information (in bits) provided by the blood tests that favour (or disfavour) the corresponding disease.

1 $$r\,(x)=\,\mathrm{log}\,\frac{predicted\,(x)}{prevalence\,(x)}=\,\mathrm{log}\,predicted\,(x)-\,\mathrm{log}\,prevalence\,(x)\,(radius)\,$$

2 $$\varphi (x)=2\pi \,predicted\,(x)\,({angle}\,in\,radians)$$

In these two equations (1, 2), *r* is the radius (information score) and *ø* the angle (predicted probability) of disease *x*. Positive radii correspond to information scores in favour of a given disease, while negative radii indicate that the blood test results reduce the possibility of a particular disease (information score is negative). Because we cannot depict negative radii, the inner circle in the graph offsets the radius zero (indifferent information). Negative radii are therefore represented inside the innermost circle.

If we look at the graph as a whole, it is strongly (although not identically) related to the Kullback-Leibler^27,28^ divergence between posttest (predicted) and pretest (prevalence) probability distributions.

This method of visualizing the results (Fig. 6) emphasizes both the most likely diseases and those with the highest information scores. To reach a final diagnosis, a physician should consider both types of salient diseases. This is especially useful because predicted probabilities may not be optimally calibrated: machine learning classifiers often tend to favour the more frequent classes (diseases) and neglect the less frequent ones.

### Data availability

Both the predictive models are available at www.smartbloodanalytics.com upon registration. The twenty clinical test cases with all the data and predicted results can be found in Supplementary S2 Data. The study was approved by the Slovenian National Medical Ethics Committee (No. 103/11/15).

## Results

### Random forest predictive models

Utilizing a random forest algorithm, we generated two different predictive models: Smart Blood Analytics Haematology 181 (SBA-HEM181), which was trained with 181 different parameters, and Smart Blood Analytics Haematology 61 (SBA-HEM061), which was trained with 61 parameters. Both training sets included 43 different disease categories. We validated both classifiers using stratified tenfold cross-validation. The SBA-HEM181 prediction accuracy was 59%.

**Figure 7:**
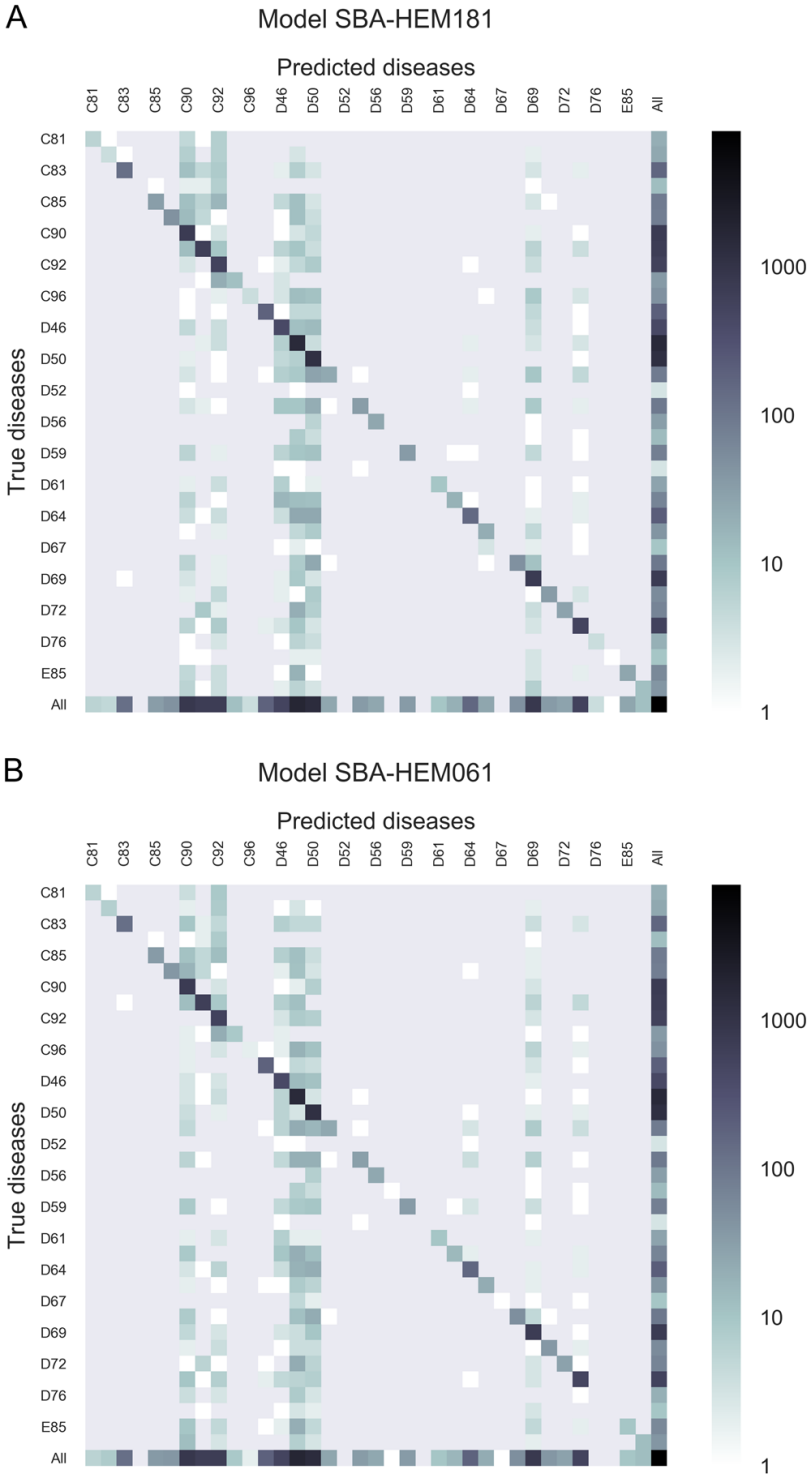
Confusion matrix for the five most likely diseases for both predictive models **(A**) SBA-HEM181 and (**B**) for SBA-HEM61. Each column of the matrix represents instances of predicted diseases, while each row represents instances of actual diseases. The frequencies are marked on a logarithmic scale.

Surprisingly, SBA-HEM061 performed on par with the SBA-HEM181 predictive model, attaining a prediction accuracy of 57%. If only the first five predictions are considered, the SBA-HEM181 and SBA-HEM061 model accuracies were 88% and 86%, respectively (Fig. 7). These results are remarkable given the small data coverage and—more importantly—the small subset of data used for predictive model building compared to the data that is typically needed for diagnosis. Table 3 lists the ten most important blood parameters (as estimated by ReliefF) and their frequencies.

**Table 3:** Ten most important blood parameters and their frequencies. It is interesting to note that all these parameters are present in almost all cases.

rank	parameter	frequency	relative frequency
1	Thrombocytes count	8141	0.99
2	Lymphocyte count	8122	0.99
3	Lymphocyte %	8159	0.99
4	Leukocyte count	8156	0.99
5	Neutrophils %	8150	0.99
6	Haematocrit	8151	0.99
7	Erythrocyte count	8152	0.99
8	Haemoglobin	8150	0.99
9	Mean Corpuscular Haemoglobin	8148	0.99
10	Age	8233	1.00

**Figure 8:**
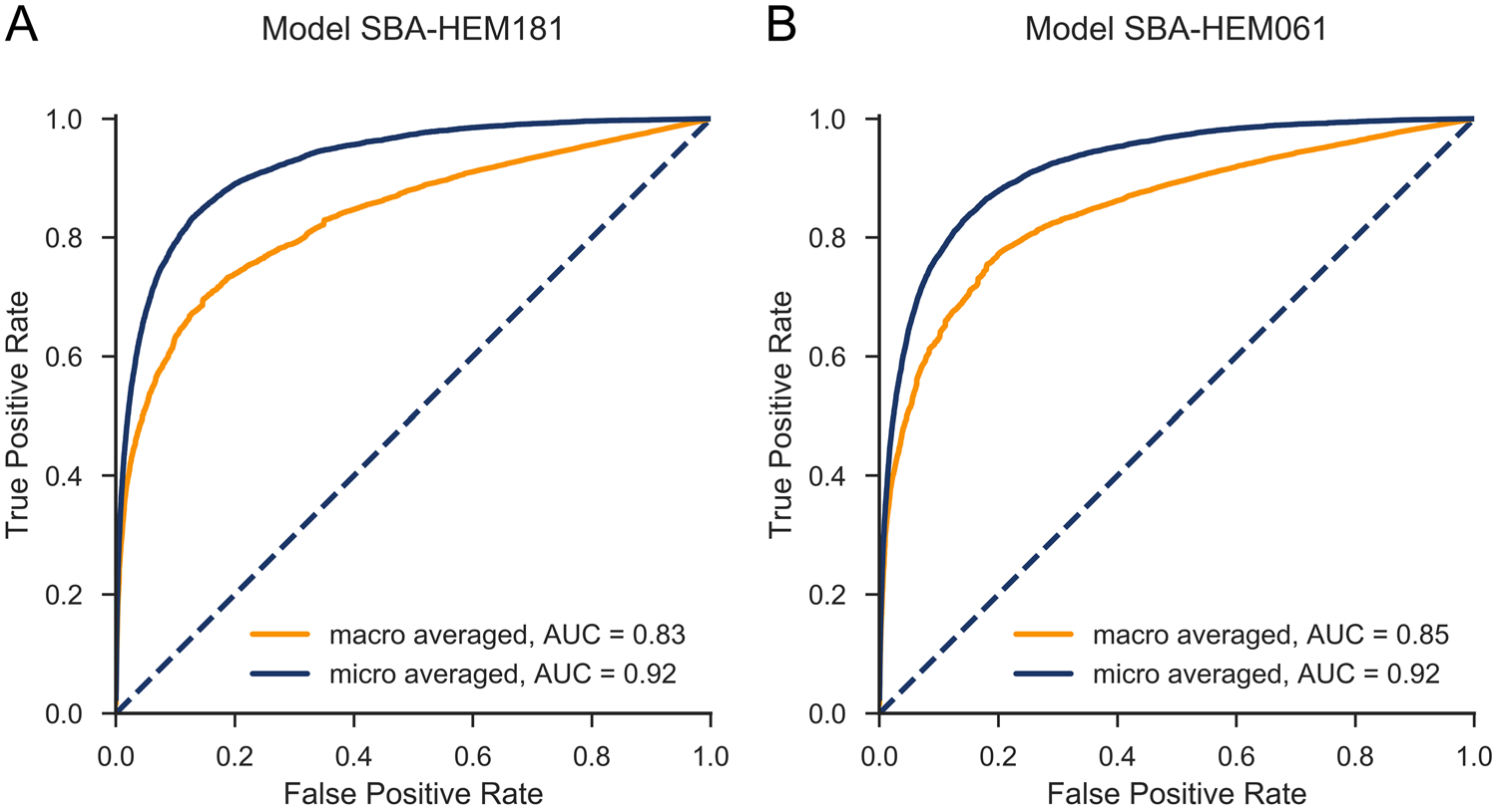
Macro- and micro-averaged ROC curves with (**A**) a full set of 181 parameters and (**B**) a basic set of 61 parameters. The curves almost overlap, and the AUCs are almost identical.

Judging from both the AUCs and the shapes of the ROC curves (Fig. 8) SBA-HEM061 performed slightly better for predicting the less prevalent diseases. Of particular interest are the differences between the macro-averaged and micro-averaged ROC curves. The micro-averaged ROC curve lies above the macro-averaged one, which indicates that both predictive models, on average, underperformed for rare diseases (Fig. 8). We tried to the improve performance on rare diseases (mostly represented by < 10 cases) by oversampling the available examples using a factor inversely proportional to their frequency. This approach not only failed to improve the performance on rare diseases, it decreased the performance on frequent diseases. Because this study did not focus on specific diseases, we did not pursue this research direction. However, if a specific need arises, we will consider testing more advanced sampling techniques such as SMOTE^29^ or ADASYN^30^.

The ROC curves for the SBA-HEM181 (Fig. 8A) and SBA-HEM061 models (Fig. 8B) are similar and exhibit almost the same AUCs, indicating that both models perform equally. We confirmed this hypothesis by applying the Wilcoxon signed rank test^31^. The null hypothesis H_0_ was that the median rankings of correct predictions for both models are significantly different. Not surprisingly, the p-values obtained on the 8,233 results using tenfold cross-validation show that we cannot reject the null hypothesis (p > 0.35). Therefore, we can conclude that the rankings of correct predictions do not differ significantly between the two models (i.e., the reduced model performs as well as the complete model).

### Model versus physician comparison

**Figure 9:**
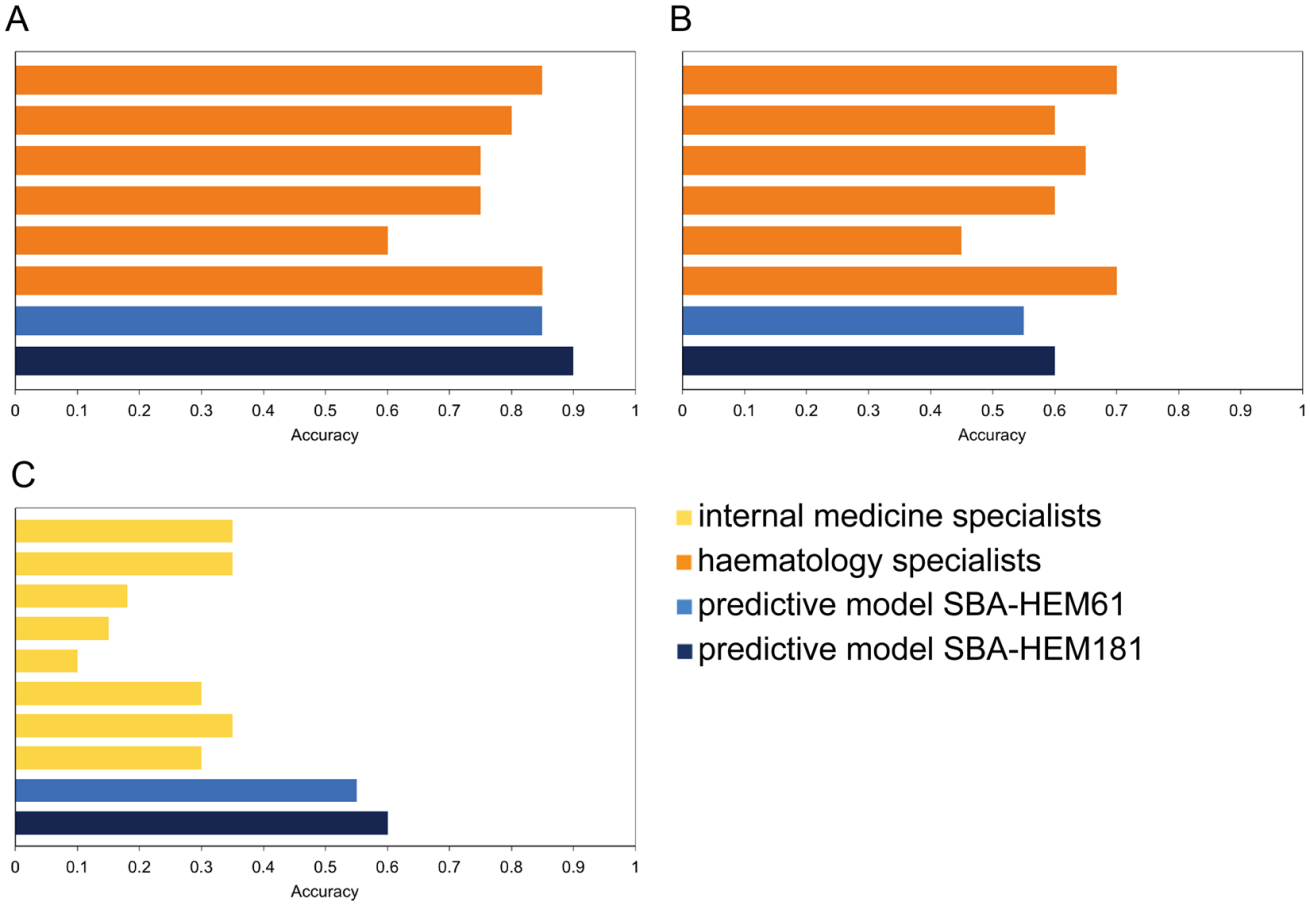
Comparison of the accuracy of internal medicine specialists with both predictive models (**A**) accuracy of the six haematology specialists compared to both predictive models when considering the five most likely predicted diseases; (**B**) accuracy of the six haematology specialists compared to both predictive models when considering only the most likely predicted disease; (**C**) accuracy of the eight non-haematology internal medicine specialists compared to both predictive models when considering the most likely predicted disease.

To compare the performance of our predictive models with that of physicians, we performed a clinical test based only on laboratory blood tests as the input data. Six haematology specialists and 8 non-haematology internal medicine specialists were presented with 20 haematological cases. Our classifier achieved an accuracy of 0.60 (0.55 SBA-HEM61), while the haematology specialists achieved an accuracy of 0.62 (Fig. 9B) and the non-haematology internal medicine specialists achieved an accuracy of 0.26(Fig. 9C). When considering only the first five predicted diseases, the accuracy of the prediction model SBA-HEM181 increases to 0.90 (0.85 for SBA-HEM61) and that of the haematology specialists increases to 0.77 (Fig. 9A). The Internal medicine specialists predicted only one possible disease.

To further compare the physicians’ results with the results from our models, we conducted Wilcoxon signed rank tests^31^. The statistical tests were performed on paired samples of 20 results—one set from the physicians and one from either of the predictive models (SBA-HEM181 or SBA-HEM61). The null hypothesis was that the results (median ranks of correct predictions) of the haematology specialists and that of the models do not differ significantly. For both SBA-HEM181 and SBA-HEM61 the Wilcoxon signed-rank test established that there is no significant difference (p > 0.05) between either of the models and the haematology specialists. However, when compared with the internal medicine specialists, the test results were significant (p < 0.01) in favour of the predictive models, which perform better.

### Illustrative example

A 65-year old man who had been exhibiting diffuse abdominal pain, tiredness, weight loss, back pain and spontaneous bruising over the last 9 months was admitted to the UMCL for hormonal evaluation of an adrenal tumour revealed by computer tomography five months earlier. The standard hormonal evaluation excluded the adrenal tumour’s hormonal autonomy, but serum protein evaluation revealed increased serum free light chains, indicating a plasma cell disorder. A bone marrow biopsy was performed, and the patient was discharged without diagnosis or treatment while waiting for the bone marrow histology results. After discharge, the laboratory results were analysed on the Smart Blood Analytics website, where the SBA-HEM181 model proposed plasmocytoma (ICD category code D90) as the second most likely disease using only the admission laboratory results and as the most likely haematological disease using all the patients laboratory results during hospitalization (Fig. 6). His “normal” laboratory results from two years earlier were also analysed, and the SBA-HEM181 model proposed plasmocytoma as the fourth most likely disease at that time. One month after admission, the patient received confirmation from a haematology specialist that he had plasmocytoma and amyloidosis (ICD category code E85).

This example demonstrates how our model could help physicians facilitate the diagnostic procedure and, in the future, reduce the number of tests, particularly for insidious diseases such as plasmocytoma that begin several years before symptoms appear. Our model could also help patients in the process of requesting a second opinion.

## Discussion

In this study, we showed that a machine learning approach, using a random forest algorithm trained on large amounts of multianalyte sets of haematologic disease laboratory blood test results, is able to interpret the results and predict diseases with an accuracy on par with experienced haematology specialists, while outperforming internal medicine specialists by a margin of more than two. Our study includes 43 possible disease categories (classes), with a majority class prevalence of 18.4% and a Shannon entropy of 3.95 bit, which is reasonably close to the theoretical maximum of 5.42 for 43 classes. This problem is difficult for a machine learning task in the medical domain according to the UCI repository^32^, where more than 50% of medical datasets consist of only two classes and deal with a single disease (present/absent). An added difficulty was that more than 75% of the attribute (parameter) values were missing. Under these conditions, a classification (diagnostic) accuracy of 0.57 (SBA-HEM61) and 0.59 (SBA-HEM181) for the most likely disease represents excellent results; they are comparable to that of a haematology specialist and far beyond the expected accuracy scores for a simple majority (0.184) or random predictive model (0.093).

The utility of our predictive models for haematological diseases diagnosis was confirmed through a clinical test in which both predictive models were able to diagnose haematological disease types as well as experienced haematology specialists and significantly better than general internal medicine specialists. The prediction accuracies of SBA-HEM061 and SBA-HEM181, using admission blood laboratory tests, were 0.55 and 0.60, respectively, while the haematology specialists achieved an accuracy of 0.60. These three results are not significantly different (Wilcoxon signed-rank test, p > 0.05). However, when considering the five most likely diagnoses proposed by SBA-HEM181 and the haematology specialists, the accuracies of SBA-HEM181 and that of the haematology specialists improved to 0.90 and 0.77, respectively, which are significantly different (p < 0.05).

Accordingly, SBA could be used to assist physicians not specialized in haematology by facilitating the diagnostic procedure and suggesting proper and early patient referral. In addition, this study showed that many laboratory tests are not needed to arrive at a correct diagnosis; however, they may be useful for additional confirmations. The smaller predictive model SBA-HEM061, trained on only a third of the available laboratory variables (routine laboratory tests that are the most frequently used by physicians), was equally successful in predicting the most likely diseases as determined by haematology specialists (57% accuracy). This result can clearly be seen from Fig. 4. Moreover, the accuracy of SBA-HEM061 for predicting the discharge diagnoses determined by haematology specialists increased to 90%. This suggests that there is a substantial information redundancy as well as interdependency in laboratory tests because the accuracy of SBA-HEM061 in predicting diagnoses based on laboratory results was equivalent to using all 181 laboratory tests, including the 61 routinely used laboratory tests. From this we can conclude that a machine learning approach in laboratory diagnostics could aid physicians in making early diagnoses of a disease using fewer laboratory tests^9^.

The results of this study are encouraging, and they oppose established practice and the reflections of physicians. In clinical practice, a physician’s ability to quickly reach a diagnosis and determine a management plan is extraordinary, and it resembles an “art,” because much of the process involves skills in clinical decision-making—the essence of which most physicians find difficult to describe in an actionable manner. This view is further reinforced by a study showing that patient histories, physical examinations and laboratory investigations lead to a final diagnosis in 76%, 10% and 11% of the cases, respectively^33^. Accordingly, most physicians are convinced that laboratory test results, viewed in isolation, are typically of limited diagnostic value in making a differential diagnosis, especially in situations where medical knowledge and experience play a significant role^10^. Such an opinion is understandable, considering the absence of published articles on advanced analytical and machine learning approaches in blood laboratory diagnostics. Therefore, it would be useful for physicians to use a machine learning approach in the interpretation of blood laboratory test results. Instead of ordering an increasing number of unnecessary and costly tests, physicians could improve their interpretations of the test data by using clinical decision support systems based on machine learning approaches. These machine learning models could help physicians interpret the multianalyte sets of many individual blood laboratory test results. This is important, because it seems that physicians use even individual laboratory tests insufficiently. In 15% of cases, physicians admitted to not completely understanding what tests they were ordering and 25% admitted to being confused by the returned results^34^. The sheer volume of available tests and their rapid rate of increase reduce the likelihood of a physicians’ proper understanding and use of the results to improve the outcome.

Machine learning to interpret blood laboratory tests could be particularly useful for general practitioners and other physicians unfamiliar with detailed haematological diagnostics, because our models predict the most likely haematological diseases using only a patients’ common blood laboratory results. This capability could also help physicians refer patients correctly. The usefulness of such models would be greatly improved if they were to become an integral part of medical information systems, i.e., as decision support for health care providers that spontaneously included a list of the most likely diseases in laboratory reports. Moreover, such integration could stimulate the production of consolidated, large-scale, digital databases of patient information that, by themselves, could change the course of medicine.

In a time when patients are becoming increasingly aware and want to be actively involved in their treatment, patients and their relatives could use a diagnostic tool like ours in the process of requesting a second opinion. Thus, the user-friendly Smart Blood Analytics website (www.smartbloodanalytics.com) with numerical and graphical representations of proposed diseases was developed and tested with actual patients. The most interesting example was our illustrative case, in which our model was able to predict the correct disease from laboratory results obtained several years prior to the occurrence of the existing disease and its symptoms.

The excellent results obtained in this study has encouraged further work to apply of machine learning to the wider field of internal medicine. Models built for other fields of internal medicine also show promising results.

## Conclusions

Every disease originates from or causes changes on a cellular and molecular level, and these changes are almost always directly or indirectly detectable through changes in blood parameter values. These changes can be large, and physicians can observe them by checking for blood parameter values outside of normal ranges. However, small changes and/or interactions between multiple different blood parameters, which are equally important for detecting pathological patterns (disease “fingerprints”), can be easily overlooked. The findings of this study imply that the value of blood test results is often underestimated. Furthermore, machine learning models can recognize disease-related blood laboratory patterns that are beyond current medical knowledge, resulting in higher diagnostic accuracy compared to traditional quantitative interpretations based on reference ranges for blood parameters. Adopting a machine learning approach in blood laboratory-based diagnosis could lead to a fundamental change in differential diagnosis and result in the modification of currently accepted guidelines. Nevertheless, physicians will always be needed to interact with patients^4^ and should retain access to all raw laboratory data.

In conclusion, a machine learning approach using a random forest algorithm with a sufficiently large data set indicates the presence of both substantial information redundancy and an unobserved potential of laboratory blood test results for diagnosing disease. SBA predictive models show great promise in medical laboratory diagnoses and could not only be of considerable value to both physicians and patients but also have widespread beneficial impacts on healthcare costs.

## Electronic supplementary material

Supplementary Information
