# Supplementary material for: An application of machine learning to haematological diagnosis
Source: Sci Rep. 2018 Jan 11;8:411. doi: 10.1038/s41598-017-18564-8 (PMC5765139; doi:10.1038/s41598-017-18564-8)
Supplement: Supplementary file 1 — Supplementary Information [file 41598_2017_18564_MOESM1_ESM.pdf]

An application of machine learning to haematological diagnosis

Gregor Gunčar[1,¶], Matjaž Kukar[1, ¶], Mateja Notar[1], Miran Brvar[2], Peter Černelč[3], Manca Notar[1], Marko Notar[1,*]

Supplementary Information

Supplementary Table S1. Basic set of 61 parameters used for SBA-HEM061 predictive model

parameter name
Age
Alanine Aminotransferase, ALT, GPT
Alkaline Phosphatase, AP
Aspartate Aminotransferase, AST, GOT
Band neutrophil %
Basophils %
Basophils count
Bilirubin Direct
Bilirubin, Total
Blast cells %
C-Reactive Protein, CRP
Calcium, Ca
Carbon dioxide content, arterial blood
Chloride, Cl
Creatinine
Eosinophils %
Eosinophils count
Erythroblast %
Erythroblasts count
Erythrocyte count, RBC
Erythrocyte Distribution Width, RDW
Ferritin
Gamma-Glutamyltransferase, GGT
Glucose
Hematocrit
Hemoglobin
Hypochromic red cells %, Hypo-He
Immature Granulocytes %
Immature granulocytes count
Iron-binding capacity, total
Iron, Fe
Lactate Dehydrogenase, LD
Leukocyte count, WBC
Lymphocyte %
Lymphocyte count
Mean Corpuscular Hemoglobin Concentration, MCHC
Mean Corpuscular Hemoglobin, MCH
Mean Corpuscular Volume, MCV
Mean Platelet Volume, MPV
Metamyelocytes %
Monocyte %
Monocyte count
Myelocytes %
Neutrophils %
Neutrophils count
Phosphate
Platelet distribution width, PDW
Potassium, K
Promonocytes %
Promyelocyte %
Proteins, total
Reticulocyte hemoglobin equivalent, Ret-He
Reticulocytes %
Reticulocytes count, urgent automated
Sex
Sodium, Na
SPE Albumin
Thrombocytes count, Platelet count
Transferrin saturation %
Urea
Uric acid

Supplementary Data S2. Twenty cases used in clinical test with all laboratory blood tests and SBA-HEM181 predictive model results

Smart Blood Analytics report #605

#68 primer 1 Male,

Blood Parameters

Lab Test Date: /

Hematology

		Value	Unit	Reference range
Leukocyte count, WBC	L	2.99	1E9/L	4-10
Neutrophils count	L	0.87	1E9/L	1.5-7.4
Lymphocyte count		1.82	1E9/L	1-3.4
Monocyte count	L	0.18	1E9/L	0.21-0.92
Eosinophils count		0.09	1E9/L	0.02-0.67
Basophils count		0	1E9/L	0-0.13
Immature granulocytes count	H	0.03	1E9/L	0-0
Erythroblasts count		0	1E9/L	0-0
Neutrophils %	L	0.29	1	0.4-0.7
Lymphocyte %	H	0.61	1	0.22-0.44
Monocyte %		0.06	1	0.04-0.11
Eosinophils %		0.03	1	0-0.08
Basophils %		0	1	0-0.03
Immature Granulocytes %		0.003	1	0-0.01
Erythroblast %	H	0.01	1	0-0
Hemoglobin	L	76	g/L	130-170
Hematocrit	L	0.215	1	0.4-0.5
Erythrocyte count, RBC	L	1.89	1E12/L	4.5-5.9
Mean Corpuscular Volume, MCV	H	113.8	fL	83-101
Mean Corpuscular Hemoglobin, MCH	H	40.2	pg/cell	27-32
Mean Corpuscular Hemoglobin Concentration, MCHC	H	353	g/L	315-345
Erythrocyte Distribution Width, RDW	H	0.146	1	0.116-0.14
Reticulocytes count, urgent automated		24.9	1E9/L	20-100
Reticulocytes %		0.0132	1	0.005-0.025
Hypochromic red cells %, Hypo-He		2	%	0-2.7
Thrombocytes count, Platelet count	L	20	1E9/L	150-410
Blast cells %		0	1	0-0.05
Promonocytes %		0	1	0-0
Myelocytes %		0	1	0-0
Metamyelocytes %	L	0.01	1	0.13-0.32
Band neutrophil %		0	1	0-0.1

Blood gas analysis and hemoglobins

		Value	Unit	Reference range
Reticulocyte hemoglobin equivalent, Ret-He	H	35.8	pg	28-35
Carbon dioxide content, arterial blood		28	mmol/L	24-32

Biochemistry

		Value	Unit	Reference range
Glucose	H	6.1	mmol/L	4.1-5.6
Urea		6.1	mmol/L	2.1-7.1
Potassium, K		4.5	mmol/L	3.5-5
Sodium, Na		141	mmol/L	136-145
Chloride, Cl	H	107	mmol/L	98-106
Calcium, Ca		2.34	mmol/L	2.15-2.5
Phosphate	L	0.74	mmol/L	0.87-1.45
Creatinine		73	µmol/L	0-133
Uric acid		258	µmol/L	150-480
Bilirubin, Total	L	5	µmol/L	5.1-17
Bilirubin Direct		2	µmol/L	1.7-5.1
Iron, Fe		25.1	µmol/L	11.6-31.3
Iron-binding capacity, total		49.7	µmol/L	40.8-76.7

Protein and amino acid analysis

		Value	Unit	Reference range
Proteins, total		73	g/L	63-79
SPE Albumin		45	g/L	35-50
Transferrin saturation %	H	0.505	1	0.2-0.5
Ferritin	H	387	µg/L	30-300
C-Reactive Protein, CRP	H	10	mg/L	0-5

Enzymes

		Value	Unit	Reference range
Alkaline Phosphatase, AP	L	0.84	µkat/L	0.9-2.18
Aspartate Aminotransferase, AST, GOT		0.34	µkat/L	0.17-1
Alanine Aminotransferase, ALT, GPT		0.36	µkat/L	0.22-0.68

Plasma cells %	0 1	0-0	Gamma-Glutamyltransferase, GGT		0.29 µkat/L	0-0.94
			Lactate Dehydrogenase, LD	H	4.07 µkat/L	1.7-3.2

Smart Blood Analytics Report

Model used: Hematology (CT181)

Report Date: 2017/05/30

	ICD	Prevalence	Prediction	Information	Description
	D46	5.56%	36.20%	2.70	**Myelodysplastic syndromes**
	D75	6.64%	15.80%	1.25	**Other diseases of blood and blood-forming organs**
	C92	7.01%	8.20%	0.23	**Myeloid leukaemia**
	D64	2.65%	6.00%	1.18	**Other anaemias**
	C91	8.45%	5.60%	-0.59	**Lymphoid leukaemia**
	D53	1.14%	4.40%	1.94	**Other nutritional anaemias**
	D69	9.02%	3.40%	-1.41	**Purpura and other haemorrhagic conditions**
	D61	0.34%	3.00%	3.14	**Other aplastic anaemias**
	C90	8.99%	2.60%	-1.79	**Multiple myeloma and malignant plasma cell neoplasms**
	C83	2.02%	2.00%	-0.01	**Diffuse non-Hodgkin lymphoma**

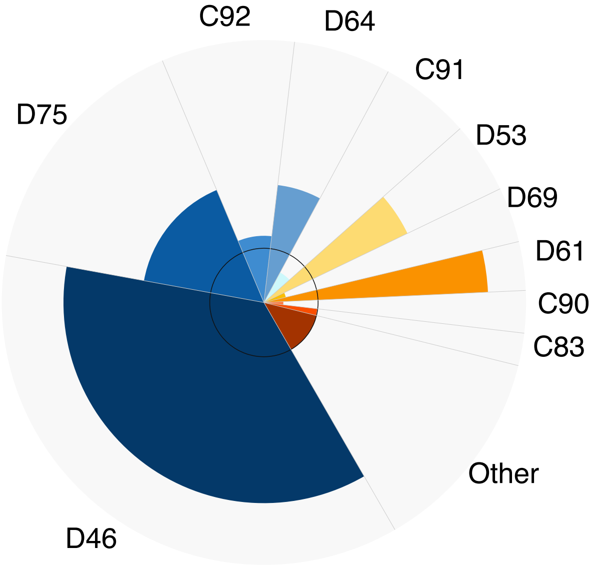
C92
D64
C91
D75
D53
D69
D61
C90
C83
Other
D46

Smart Blood Analytics report #606

#69 primer 2 Male,

Blood Parameters

Lab Test Date: /

Hematology

	Flag	Value	Unit	Reference range
Leukocyte count, WBC	L	1.39	1E9/L	4-10
Neutrophils count	L	0.06	1E9/L	1.5-7.4
Lymphocyte count		1.31	1E9/L	1-3.4
Monocyte count	L	0.01	1E9/L	0.21-0.92
Eosinophils count	L	0.01	1E9/L	0.02-0.67
Basophils count		0	1E9/L	0-0.13
Immature granulocytes count		0	1E9/L	0-0
Erythroblasts count		0	1E9/L	0-0
Neutrophils %	L	0.04	1	0.4-0.7
Lymphocyte %	H	94	%	22-44
Monocyte %	L	1	%	4-11
Eosinophils %		1	%	0-8
Basophils %		0	%	0-3
Immature Granulocytes %		0	%	0-1
Erythroblast %		0	%	0-0
Hemoglobin	L	46	g/L	130-170
Hematocrit	L	0.131	1	0.4-0.5
Erythrocyte count, RBC	L	1.26	1E6/µL	4.5-5.9
Mean Corpuscular Volume, MCV	H	104	fL	83-101
Mean Corpuscular Hemoglobin, MCH	H	36.5	pg/cell	27-32
Mean Corpuscular Hemoglobin Concentration, MCHC	H	351	g/L	315-345
Erythrocyte Distribution Width, RDW		13.2	%	11.6-14
Thrombocytes count, Platelet count	L	14	1E9/L	150-410
Blast cells %		0	1	0-0.05
Promonocytes %		0	1	0-0
Myelocytes %		0	1	0-0
Metamyelocytes %	L	0	1	0.13-0.32
Band neutrophil %		0	1	0-0.1
Plasmacytes %		0	1	0-0

Biochemistry

	Flag	Value	Unit	Reference range
Glucose	H	5.7	mmol/L	4.1-5.6
Urea	H	10.6	mmol/L	2.1-7.1
Sodium, Na		136	mmol/L	136-145
Chloride, Cl		100	mmol/L	98-106
Calcium, Ca	L	2.06	mmol/L	2.15-2.5
Phosphate		1.18	mmol/L	0.87-1.45
Creatinine		91	µmol/L	0-133
Uric acid		217	µmol/L	150-480
Iron, Fe		20.2	µmol/L	11.6-31.3
Iron-binding capacity, total		43.5	µmol/L	40.8-76.7

Blood gas analysis and hemoglobins

	Flag	Value	Unit	Reference range
Carbon dioxide content, arterial blood	L	23	mmol/L	24-32

Protein and amino acid analysis

	Flag	Value	Unit	Reference range
Proteins, total		65	g/L	63-79
SPE Albumin		39	g/L	35-50
Transferrin saturation %		0.464	1	0.2-0.5
Ferritin	H	511	µg/L	30-300
C-Reactive Protein, CRP	H	85	mg/L	0-5

Enzymes

	Flag	Value	Unit	Reference range
Alkaline Phosphatase, AP		1.70	µkat/L	0.9-2.18
Aspartate Aminotransferase, AST, GOT		0.53	µkat/L	0.17-1
Alanine Aminotransferase, ALT, GPT		0.49	µkat/L	0.22-0.68
Gamma-Glutamyltransferase, GGT		0.73	µkat/L	0-0.94

Smart Blood Analytics Report

Model used: Hematology (CT181)

Report Date: 2017/05/30

	ICD	Prevalence	Prediction	Information	Description
	C92	7.01%	41.20%	2.56	**Myeloid leukaemia**
	D46	5.56%	17.40%	1.65	**Myelodysplastic syndromes**
	D75	6.64%	10.60%	0.67	**Other diseases of blood and blood-forming organs**
	C91	8.45%	5.20%	-0.70	**Lymphoid leukaemia**
	D61	0.34%	4.80%	3.82	**Other aplastic anaemias**
	C93	0.45%	4.20%	3.22	**Monocytic leukaemia**
	C90	8.99%	3.80%	-1.24	**Multiple myeloma and malignant plasma cell neoplasms**
	D53	1.14%	3.20%	1.48	**Other nutritional anaemias**
	C94	0.11%	2.20%	4.33	**Other leukaemias of specified cell type**
	D64	2.65%	1.40%	-0.92	**Other anaemias**

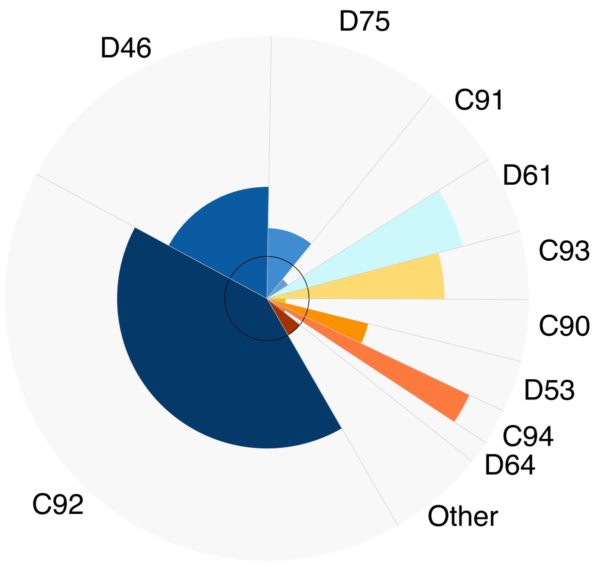
D75
D46
C91
D61
C93
C90
D53
C94
D64
Other
C92

Smart Blood Analytics report #607

#70 primer 3 — Male,

Blood Parameters

Lab Test Date: /

Hematology

		Value	Unit	Reference range
Leukocyte count, WBC	H	11	1E9/L	4-10
Neutrophils count	H	8.69	1E9/L	1.5-7.4
Lymphocyte count		2.20	1E9/L	1-3.4
Monocyte count	L	0	1E9/L	0.21-0.92
Eosinophils count	L	0	1E9/L	0.02-0.67
Basophils count		0.09	1E9/L	0-0.13
Immature granulocytes count		0	1E9/L	0-0
Erythroblasts count		0	1E9/L	0-0
Neutrophils %	H	0.78	1	0.4-0.7
Lymphocyte %	L	0.20	1	0.22-0.44
Monocyte %	L	0	1	0.04-0.11
Basophils %		0.01	1	0-0.03
Immature Granulocytes %		0.005	1	0-0.01
Hemoglobin	L	121	g/L	130-170
Hematocrit	L	0.392	1	0.4-0.5
Erythrocyte count, RBC	H	5.93	1E12/L	4.5-5.9
Mean Corpuscular Volume, MCV	L	66.1	fL	83-101
Mean Corpuscular Hemoglobin, MCH	L	20.4	pg/cell	27-32
Mean Corpuscular Hemoglobin Concentration, MCHC	L	309	g/L	315-345
Erythrocyte Distribution Width, RDW	H	0.16	1	0.116-0.14
Reticulocytes count, urgent automated		82.4	1E9/L	20-100
Reticulocytes %		0.0139	1	0.005-0.025
Hypochromic red cells %, Hypo-He	H	0.109	1	0-0.027
Thrombocytes count, Platelet count		368	1E9/L	150-410
Mean Platelet Volume, MPV		9.6	fL	9.4-12.2
Platelet distribution width, PDW	L	10.9		11-16.9
Blast cells %		0	1	0-0.05
Promonocytes %		0	1	0-0
Myelocytes %		0	1	0-0
Metamyelocytes %	L	0	1	0.13-0.32
Band neutrophil %		0.01	1	0-0.1

Blood gas analysis and hemoglobins

		Value	Unit	Reference range
Reticulocyte hemoglobin equivalent, Ret-He	L	21.1	pg	28-35
Carbon dioxide content, arterial blood	H	33	mmol/L	24-32

Biochemistry

		Value	Unit	Reference range
Glucose		4.5	mmol/L	4.1-5.6
Urea		4.3	mmol/L	2.1-7.1
Potassium, K	H	5.50	mmol/L	3.5-5
Sodium, Na		143	mmol/L	136-145
Chloride, Cl	H	107	mmol/L	98-106
Calcium, Ca		2.15	mmol/L	2.15-2.5
Phosphate	L	0.85	mmol/L	0.87-1.45
Creatinine		62	µmol/L	0-133
Uric acid		313	µmol/L	150-480
Bilirubin, Total		7	µmol/L	5.1-17
Bilirubin Direct		3	µmol/L	1.7-5.1
Iron, Fe		19.6	µmol/L	11.6-31.3
Iron-binding capacity, total		62.1	µmol/L	40.8-76.7

Protein and amino acid analysis

		Value	Unit	Reference range
Proteins, total		66	g/L	63-79
SPE Albumin		42	g/L	35-50
Transferrin saturation %		0.316	1	0.2-0.5
Ferritin		51	µg/L	30-300
C-Reactive Protein, CRP		4	mg/L	0-5

Enzymes

		Value	Unit	Reference range
Alkaline Phosphatase, AP		1.57	µkat/L	0.9-2.18
Aspartate Aminotransferase, AST, GOT		0.36	µkat/L	0.17-1
Alanine Aminotransferase, ALT, GPT		0.39	µkat/L	0.22-0.68

Eosinophils %	0 1	0-0.08	Gamma-Glutamyltransferase, GGT		0.26 µkat/L	0-0.94
Plasmacytes %	0 1	0-0	Lactate Dehydrogenase, LD	H	3.41 µkat/L	1.7-3.2

Smart Blood Analytics Report

Model used: Hematology (CT181)

Report Date: 2017/05/30

	ICD	Prevalence	Prediction	Information	Description
	D50	14.45%	26.60%	0.88	**Iron deficiency anaemia**
	D56	0.39%	17.40%	5.48	**Thalassaemia**
	D47	18.50%	16.20%	-0.19	**Other neoplasms of uncertain or unknown behaviour of lymphoid, haematopoietic and related tissue**
	D45	2.47%	9.80%	1.99	**Polycythaemia vera**
	D69	9.02%	5.20%	-0.80	**Purpura and other haemorrhagic conditions**
	D64	2.65%	4.00%	0.59	**Other anaemias**
	D75	6.64%	3.80%	-0.81	**Other diseases of blood and blood-forming organs**
	D68	1.20%	3.00%	1.32	**Other coagulation defects**
	D46	5.56%	2.60%	-1.10	**Myelodysplastic syndromes**
	D72	0.85%	1.40%	0.72	**Other disorders of white blood cells**

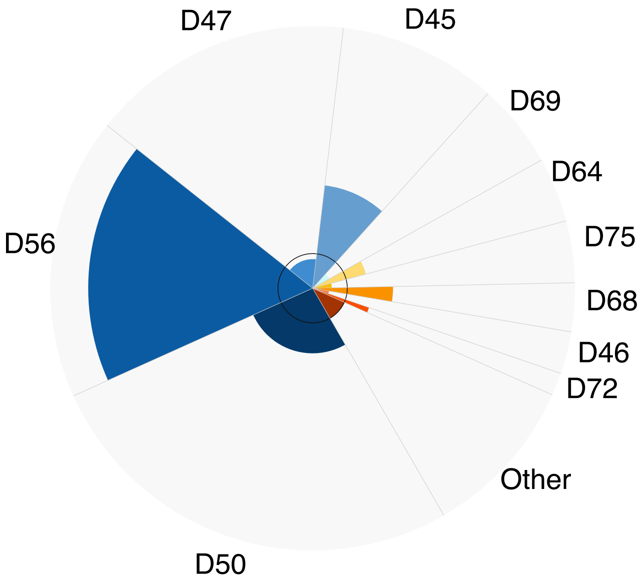
D47
D45
D69
D64
D75
D68
D46
D72
Other
D50
D56

Smart Blood Analytics report #609

#71 primer 4 — Female,

Blood Parameters

Lab Test Date: /

Hematology

		Value	Unit	Reference range
Leukocyte count, WBC	L	3.99	1E9/L	4-10
Neutrophils count		2.63	1E9/L	1.5-7.4
Lymphocyte count		1	1E9/L	1-3.4
Monocyte count		0.28	1E9/L	0.21-0.92
Eosinophils count		0.04	1E9/L	0.02-0.67
Basophils count		0.04	1E9/L	0-0.13
Immature granulocytes count		0	1E9/L	0-0
Erythroblasts count		0	1E9/L	0-0
Neutrophils %		0.66	1	0.4-0.7
Lymphocyte %		0.25	1	0.22-0.44
Monocyte %		0.07	1	0.04-0.11
Eosinophils %		0.01	1	0-0.08
Basophils %		0.01	1	0-0.03
Immature Granulocytes %		0	1	0-0.01
Hemoglobin		128	g/L	120-150
Hematocrit		0.391	1	0.36-0.46
Erythrocyte count, RBC		4.22	1E12/L	4-5.2
Mean Corpuscular Volume, MCV		92.7	fL	83-101
Mean Corpuscular Hemoglobin, MCH		30.3	pg/cell	27-32
Mean Corpuscular Hemoglobin Concentration, MCHC		327	g/L	315-345
Erythrocyte Distribution Width, RDW		0.135	1	0.116-0.14
Thrombocytes count, Platelet count		316	1E9/L	150-410
Mean Platelet Volume, MPV		10	fL	9.2-12.9
Platelet distribution width, PDW	L	10.5		11-16.9
Blast cells %		0	1	0-0.05
Promonocytes %		0	1	0-0
Myelocytes %		0	1	0-0
Metamyelocytes %	L	0	1	0.13-0.32
Band neutrophil %		0	1	0-0.1
Plasmacytes %		0	1	0-0
Erythroblast %		0	1	0-0

Biochemistry

	Value	Unit	Reference range
Glucose	4.8	mmol/L	4.1-5.6
Urea	4.1	mmol/L	2.1-7.1
Potassium, K	4.5	mmol/L	3.5-5
Sodium, Na	138	mmol/L	136-145
Chloride, Cl	100	mmol/L	98-106
Calcium, Ca	2.39	mmol/L	2.15-2.5
Phosphate	0.92	mmol/L	0.87-1.45
Creatinine	60	µmol/L	0-133
Uric acid	182	µmol/L	90-360
Bilirubin, Total	11	µmol/L	5.1-17
Bilirubin Direct	4	µmol/L	1.7-5.1
Iron, Fe	24.2	µmol/L	9-30.4
Iron-binding capacity, total	57.1	µmol/L	40.8-76.7

Blood gas analysis and hemoglobins

	Value	Unit	Reference range
Carbon dioxide content, arterial blood	28	mmol/L	24-32

Protein and amino acid analysis

	Value	Unit	Reference range
Proteins, total	73	g/L	63-79
SPE Albumin	44	g/L	35-50
Transferrin saturation %	0.42	1	0.2-0.5
Ferritin	57	µg/L	10-200
C-Reactive Protein, CRP	4	mg/L	0-5

Enzymes

	Value	Unit	Reference range
Alkaline Phosphatase, AP	0.81	µkat/L	0.71-1.67
Aspartate Aminotransferase, AST, GOT	0.28	µkat/L	0.17-1
Alanine Aminotransferase, ALT, GPT	0.27	µkat/L	0.17-0.48
Gamma-Glutamyltransferase, GGT	0.20	µkat/L	0-0.65
Lactate Dehydrogenase, LD	2.43	µkat/L	1.7-3.2

Smart Blood Analytics Report

Model used: Hematology (CT181)

Report Date: 2017/05/30

	ICD	Prevalence	Prediction	Information	Description
	D50	14.45%	39.60%	1.45	**Iron deficiency anaemia**
	D47	18.50%	8.80%	-1.07	**Other neoplasms of uncertain or unknown behaviour of lymphoid, haematopoietic and related tissue**
	D75	6.64%	8.80%	0.41	**Other diseases of blood and blood-forming organs**
	D68	1.20%	7.40%	2.62	**Other coagulation defects**
	D69	9.02%	6.60%	-0.45	**Purpura and other haemorrhagic conditions**
	D70	0.68%	3.80%	2.48	**Agranulocytosis**
	C92	7.01%	2.60%	-1.43	**Myeloid leukaemia**
	C96	0.57%	2.60%	2.19	**Other and unspecified malignant neoplasms of lymphoid, haematopoietic and related tissue**
	D72	0.85%	2.40%	1.50	**Other disorders of white blood cells**
	D46	5.56%	2.00%	-1.47	**Myelodysplastic syndromes**

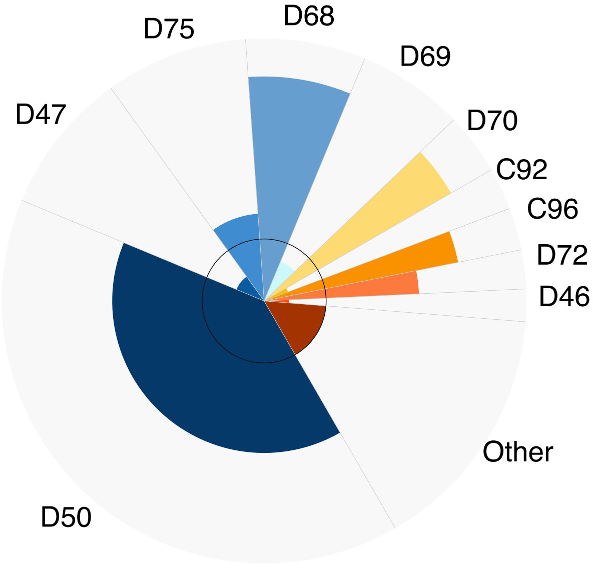
D75
D68
D69
D47
D70
C92
C96
D72
D46
Other
D50

Smart Blood Analytics report #610

#72 primer 5 Male,

Blood Parameters

Lab Test Date: /

Hematology

		Value	Unit	Reference range
Leukocyte count, WBC	H	20.93	1E9/L	4-10
Neutrophils count	H	13.60	1E9/L	1.5-7.4
Lymphocyte count		2.93	1E9/L	1-3.4
Monocyte count	H	3.14	1E9/L	0.21-0.92
Eosinophils count	L	0	1E9/L	0.02-0.67
Basophils count		0.08	1E9/L	0-0.13
Immature granulocytes count	H	1.26	1E9/L	0-0
Erythroblasts count	H	5.87	1E9/L	0-0
Neutrophils %		0.63	1	0.4-0.7
Lymphocyte %	L	0.14	1	0.22-0.44
Monocyte %	H	0.15	1	0.04-0.11
Eosinophils %		0	1	0-0.08
Basophils %		0	1	0-0.03
Immature Granulocytes %	H	0.129	1	0-0.01
Erythroblast %	H	0.27	1	0-0
Hemoglobin	L	78	g/L	130-170
Hematocrit	L	0.235	1	0.4-0.5
Erythrocyte count, RBC	L	2.44	1E12/L	4.5-5.9
Mean Corpuscular Volume, MCV		96.3	fL	83-101
Mean Corpuscular Hemoglobin, MCH		32	pg/cell	27-32
Mean Corpuscular Hemoglobin Concentration, MCHC		332	g/L	315-345
Erythrocyte Distribution Width, RDW	H	0.216	1	0.116-0.14
Thrombocytes count, Platelet count	L	23	1E9/L	150-410
Blast cells %		0	1	0-0.05
Promyelocyte %	L	0	1	0.01-0.08
Myelocytes %	H	0.02	1	0-0
Metamyelocytes %	L	0.04	1	0.13-0.32
Band neutrophil %		0.02	1	0-0.1
Promonocytes %		0	1	0-0
Plasmacytes %		0	1	0-0
Schistocyte, fragmented RBC /1000 RBC	H	0.073	/1000 Erci	-

Biochemistry

		Value	Unit	Reference range
Glucose	H	9.4	mmol/L	4.1-5.6
Urea	H	14.7	mmol/L	2.1-7.1
Potassium, K		3.80	mmol/L	3.5-5
Sodium, Na		144	mmol/L	136-145
Chloride, Cl		102	mmol/L	98-106
Calcium, Ca		2.31	mmol/L	2.15-2.5
Phosphate	H	1.59	mmol/L	0.87-1.45
Magnesium, Mg		0.84	mmol/L	0.74-1.23
Creatinine		124	µmol/L	0-133
Uric acid		429	µmol/L	150-480
Bilirubin, Total	H	18	µmol/L	5.1-17
Bilirubin Direct	H	8	µmol/L	1.7-5.1
Iron, Fe		12.2	µmol/L	11.6-31.3

Blood gas analysis and hemoglobins

		Value	Unit	Reference range
Carbon dioxide content, arterial blood	L	0.33	mmol/L	24-32

Protein and amino acid analysis

		Value	Unit	Reference range
Proteins, total	L	59	g/L	63-79
SPE Albumin	H	56	g/L	35-50
C-Reactive Protein, CRP	H	8	mg/L	0-5

Enzymes

		Value	Unit	Reference range
Alkaline Phosphatase, AP		0.94	µkat/L	0.9-2.18
Aspartate Aminotransferase, AST, GOT		0.49	µkat/L	0.17-1
Alanine Aminotransferase, ALT, GPT		0.51	µkat/L	0.22-0.68
Gamma-Glutamyltransferase, GGT		0.65	µkat/L	0-0.94
Lactate Dehydrogenase, LD	H	4.76	µkat/L	1.7-3.2

Hormones and growth factors

		Value	Unit	Reference range
NT-Pro B-Type Natriuretic Peptide	H	15386	µg/L	0-35
Procalcitonin	H	0.18	µg/L	0-0.15

Smart Blood Analytics Report

Model used: Hematology (CT181)

Report Date: 2017/05/30

ICD	Prevalence	Prediction	Information	Description
C92	7.01%	27.80%	1.99	**Myeloid leukaemia**
C90	8.99%	9.00%	0.00	**Multiple myeloma and malignant plasma cell neoplasms**
D47	18.50%	8.00%	-1.21	**Other neoplasms of uncertain or unknown behaviour of lymphoid, haematopoietic and related tissue**
D46	5.56%	7.80%	0.49	**Myelodysplastic syndromes**
C83	2.02%	6.80%	1.75	**Diffuse non-Hodgkin lymphoma**
C91	8.45%	4.60%	-0.88	**Lymphoid leukaemia**
M31	0.49%	4.60%	3.24	**Other necrotising vasculopathies**
C85	1.09%	4.40%	2.01	**Other and unspecified types of non-Hodgkin lymphoma**
D75	6.64%	3.80%	-0.81	**Other diseases of blood and blood-forming organs**
D69	9.02%	3.80%	-1.25	**Purpura and other haemorrhagic conditions**

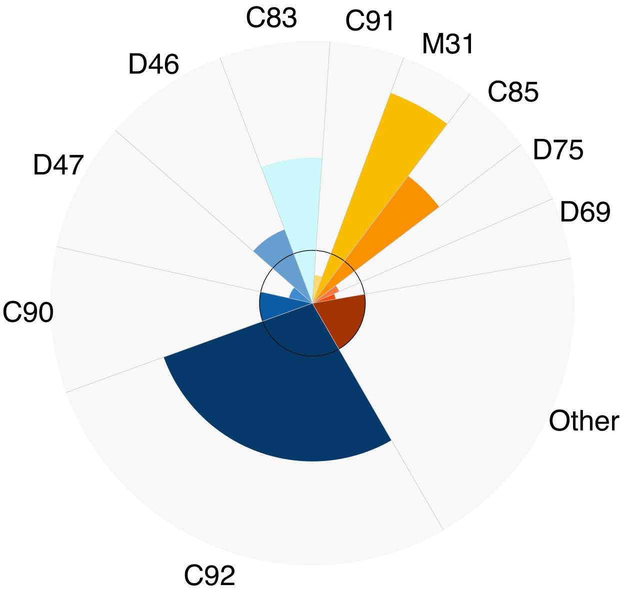
C83
C91
M31
D46
C85
D75
D47
D69
C90
Other
C92

Smart Blood Analytics report #611

#73 primer 6

Female,

Blood Parameters

Lab Test Date: /

Hematology

		Value	Unit	Reference range
Leukocyte count, WBC		5.3	1E9/L	4-10
Neutrophils count		4.08	1E9/L	1.5-7.4
Lymphocyte count	L	0.85	1E9/L	1-3.4
Monocyte count		0.26	1E9/L	0.21-0.92
Eosinophils count		0.11	1E9/L	0.02-0.67
Basophils count		0.03	1E9/L	0-0.13
Immature granulocytes count		0	1E9/L	0-0
Erythroblasts count	H	0.01	1E9/L	0-0
Neutrophils %	H	0.77	1	0.4-0.7
Lymphocyte %	L	0.16	1	0.22-0.44
Monocyte %		0.05	1	0.04-0.11
Eosinophils %		0.02	1	0-0.08
Basophils %		0	1	0-0.03
Immature Granulocytes %		0.004	1	0-0.01
Erythroblast %		0	1	0-0
Hemoglobin	L	73	g/L	120-150
Hematocrit	L	0.272	1	0.36-0.46
Erythrocyte count, RBC		4.28	1E12/L	4-5.2
Mean Corpuscular Volume, MCV	L	63.6	fL	83-101
Mean Corpuscular Hemoglobin, MCH	L	17.1	pg/cell	27-32
Mean Corpuscular Hemoglobin Concentration, MCHC	L	268	g/L	315-345
Erythrocyte Distribution Width, RDW	H	0.195	1	0.116-0.14
Reticulocytes count, urgent automated		47.5	1E9/L	20-100
Reticulocytes %		0.0111	1	0.005-0.025
Hypochromic red cells %, Hypo-He	H	0.563	1	0-0.027
Thrombocytes count, Platelet count		339	1E9/L	150-410
Mean Platelet Volume, MPV		10.1	fL	9.2-12.9
Platelet distribution width, PDW		13.2		11-16.9
Blast cells %	H	0.28	1	0-0.05
Promonocytes %		0	1	0-0
Myelocytes %		0	1	0-0

Blood gas analysis and hemoglobins

		Value	Unit	Reference range
Reticulocyte hemoglobin equivalent, Ret-He	L	17.7	pg	28-35
Carbon dioxide content, arterial blood		27	mmol/L	24-32

Biochemistry

		Value	Unit	Reference range
Glucose		5.5	mmol/L	4.1-5.6
Urea		3.9	mmol/L	2.1-7.1
Potassium, K		4.50	mmol/L	3.5-5
Sodium, Na		140	mmol/L	136-145
Chloride, Cl		106	mmol/L	98-106
Calcium, Ca		2.23	mmol/L	2.15-2.5
Phosphate		1.14	mmol/L	0.87-1.45
Creatinine		46	µmol/L	0-133
Uric acid		210	µmol/L	90-360
Bilirubin, Total		6	µmol/L	5.1-17
Bilirubin Direct		2	µmol/L	1.7-5.1
Iron, Fe		28.1	µmol/L	9-30.4
Iron-binding capacity, total		63.8	µmol/L	40.8-76.7

Protein and amino acid analysis

		Value	Unit	Reference range
Proteins, total		76	g/L	63-79
SPE Albumin		45	g/L	35-50
Transferrin saturation %		0.44	1	0.2-0.5
Ferritin	L	3	µg/L	10-200
C-Reactive Protein, CRP		4	mg/L	0-5

Enzymes

		Value	Unit	Reference range
Alkaline Phosphatase, AP		0.72	µkat/L	0.71-1.67
Alanine Aminotransferase, ALT, GPT		0.27	µkat/L	0.17-0.48
Gamma-Glutamyltransferase, GGT		0.11	µkat/L	0-0.65
Lactate Dehydrogenase, LD		2.47	µkat/L	1.7-3.2

Metamyelocytes %	L	0 1	0.13-0.32
Band neutrophil %		0 1	0-0.1
Plasmacytes %		0 1	0-0

Smart Blood Analytics Report

Model used: Hematology (CT181)

Report Date: 2017/05/30

ICD	Prevalence	Prediction	Information	Description
D50	14.45%	78.80%	2.45	**Iron deficiency anaemia**
D56	0.39%	5.20%	3.74	**Thalassaemia**
D64	2.65%	3.60%	0.44	**Other anaemias**
D47	18.50%	3.00%	-2.62	**Other neoplasms of uncertain or unknown behaviour of lymphoid, haematopoietic and related tissue**
D75	6.64%	2.40%	-1.47	**Other diseases of blood and blood-forming organs**
D46	5.56%	1.60%	-1.80	**Myelodysplastic syndromes**
D68	1.20%	1.20%	-0.00	**Other coagulation defects**
D69	9.02%	1.00%	-3.17	**Purpura and other haemorrhagic conditions**
C92	7.01%	0.80%	-3.13	**Myeloid leukaemia**
D53	1.14%	0.60%	-0.93	**Other nutritional anaemias**

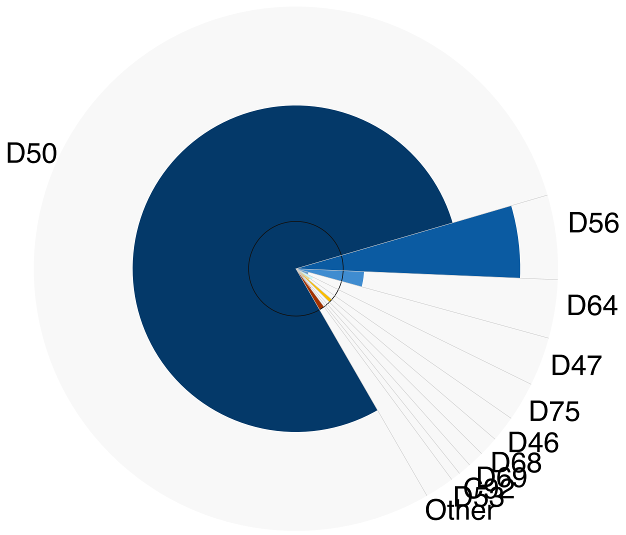
D50
D56
D64
D47
D75
D46
D68
D69
D53
Other

Smart Blood Analytics report #612

#74 primer 7 | Male,

Blood Parameters

Lab Test Date: /

Hematology

		Value	Unit	Reference range
Leukocyte count, WBC		8.94	1E9/L	4-10
Neutrophils count		6.44	1E9/L	1.5-7.4
Lymphocyte count		1.25	1E9/L	1-3.4
Monocyte count	L	0.09	1E9/L	0.21-0.92
Eosinophils count	H	1.16	1E9/L	0.02-0.67
Basophils count	H	0.16	1E9/L	0-0.13
Immature granulocytes count		0	1E9/L	0-0
Erythroblasts count		0	1E9/L	0-0
Neutrophils %	H	0.72	1	0.4-0.7
Lymphocyte %	L	0.14	1	0.22-0.44
Monocyte %	L	0.01	1	0.04-0.11
Eosinophils %	H	0.13	1	0-0.08
Basophils %		0	1	0-0.03
Immature Granulocytes %		0.002	1	0-0.01
Erythroblast %		0	1	0-0
Hemoglobin	H	171	g/L	130-170
Hematocrit	H	0.503	1	0.4-0.5
Erythrocyte count, RBC		5.83	1E12/L	4.5-5.9
Mean Corpuscular Volume, MCV		86.3	fL	83-101
Mean Corpuscular Hemoglobin, MCH		29.3	pg/cell	27-32
Mean Corpuscular Hemoglobin Concentration, MCHC		340	g/L	315-345
Erythrocyte Distribution Width, RDW	H	0.141	1	0.116-0.14
Reticulocytes count, urgent automated		84	1E9/L	20-100
Reticulocytes %		0.0144	1	0.005-0.025
Hypochromic red cells %, Hypo-He		0.0001	1	0-0.027
Thrombocytes count, Platelet count	H	817	1E9/L	150-410
Mean Platelet Volume, MPV	L	9.1	fL	9.4-12.2
Platelet distribution width, PDW	L	10.4		11-16.9
Blast cells %		0	1	0-0.05
Promyelocyte %	L	0	1	0.01-0.08
Myelocytes %		0	1	0-0

Blood gas analysis and hemoglobins

		Value	Unit	Reference range
Reticulocyte hemoglobin equivalent, Ret-He		31.5	pg	28-35
Carbon dioxide content, arterial blood		28	mmol/L	24-32

Biochemistry

		Value	Unit	Reference range
Glucose		4.1	mmol/L	4.1-5.6
Urea		5.6	mmol/L	2.1-7.1
Potassium, K	H	5.1	mmol/L	3.5-5
Sodium, Na		144	mmol/L	136-145
Chloride, Cl		101	mmol/L	98-106
Calcium, Ca		2.24	mmol/L	2.15-2.5
Phosphate		0.87	mmol/L	0.87-1.45
Creatinine		65	µmol/L	0-133
Uric acid		313	µmol/L	150-480
Bilirubin, Total	H	22	µmol/L	5.1-17
Bilirubin Direct	H	6	µmol/L	1.7-5.1
Iron, Fe		15.1	µmol/L	11.6-31.3
Iron-binding capacity, total		57.5	µmol/L	40.8-76.7

Protein and amino acid analysis

		Value	Unit	Reference range
Proteins, total		76	g/L	63-79
SPE Albumin		47	g/L	35-50
Transferrin saturation %		0.263	1	0.2-0.5
Ferritin		131	µg/L	30-300
C-Reactive Protein, CRP		4	mg/L	0-5

Enzymes

		Value	Unit	Reference range
Alkaline Phosphatase, AP		0.92	µkat/L	0.9-2.18
Aspartate Aminotransferase, AST, GOT		0.38	µkat/L	0.17-1
Alanine Aminotransferase, ALT, GPT		0.43	µkat/L	0.22-0.68

Metamyelocytes %	L	0 1	0.13-0.32	Gamma-Glutamyltransferase, GGT		0.29	µkat/L	0-0.94
Band neutrophil %		0 1	0-0.1	Lactate Dehydrogenase, LD	H	4.07	µkat/L	1.7-3.2
Promonocytes %		0 1	0-0					
Plasmacytes %		0 1	0-0					

Smart Blood Analytics Report

Model used: Hematology (CT181)

Report Date: 2017/05/30

	ICD	Prevalence	Prediction	Information	Description
	D47	18.50%	33.20%	0.84	**Other neoplasms of uncertain or unknown behaviour of lymphoid, haematopoietic and related tissue**
	D45	2.47%	27.60%	3.48	**Polycythaemia vera**
	D75	6.64%	18.00%	1.44	**Other diseases of blood and blood-forming organs**
	D72	0.85%	3.40%	2.00	**Other disorders of white blood cells**
	C96	0.57%	3.00%	2.39	**Other and unspecified malignant neoplasms of lymphoid, haematopoietic and related tissue**
	D69	9.02%	2.80%	-1.69	**Purpura and other haemorrhagic conditions**
	D50	14.45%	2.40%	-2.59	**Iron deficiency anaemia**
	C91	8.45%	1.80%	-2.23	**Lymphoid leukaemia**
	D66	0.52%	1.60%	1.61	**Hereditary factor VIII deficiency**
	C92	7.01%	1.60%	-2.13	**Myeloid leukaemia**

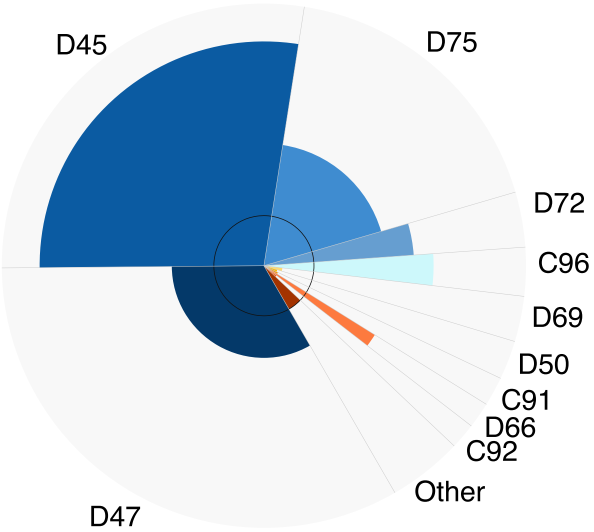
D45
D75
D72
C96
D69
D50
C91
D66
C92
Other
D47

Smart Blood Analytics report #613

#75 primer 8 — Female,

Blood Parameters

Lab Test Date: /

Hematology

	Flag	Value	Unit	Reference range
Leukocyte count, WBC		4.56	1E9/L	4-10
Neutrophils count		2.60	1E9/L	1.5-7.4
Lymphocyte count		1.19	1E9/L	1-3.4
Monocyte count		0.78	1E9/L	0.21-0.92
Eosinophils count	L	0	1E9/L	0.02-0.67
Basophils count		0.01	1E9/L	0-0.13
Immature granulocytes count		0	1E9/L	0-0
Erythroblasts count		0	1E9/L	0-0
Neutrophils %		0.57	1	0.4-0.7
Lymphocyte %		0.26	1	0.22-0.44
Monocyte %	H	0.17	1	0.04-0.11
Eosinophils %		0	1	0-0.08
Basophils %		0	1	0-0.03
Immature Granulocytes %		0.004	1	0-0.01
Erythroblast %		0	1	0-0
Hemoglobin	L	74	g/L	120-150
Hematocrit	L	0.231	1	0.36-0.46
Erythrocyte count, RBC	L	2.27	1E12/L	4-5.2
Mean Corpuscular Volume, MCV	H	101.8	fL	83-101
Mean Corpuscular Hemoglobin, MCH	H	32.6	pg/cell	27-32
Mean Corpuscular Hemoglobin Concentration, MCHC		320	g/L	315-345
Erythrocyte Distribution Width, RDW	H	0.185	1	0.116-0.14
Reticulocytes count, urgent automated	H	109	1E9/L	20-100
Reticulocytes %	H	0.048	1	0.005-0.025
Hypochromic red cells %, Hypo-He	H	0.035	1	0-0.027
Thrombocytes count, Platelet count	L	88	1E9/L	150-410
Mean Platelet Volume, MPV		12.1	fL	9.2-12.9
Platelet distribution width, PDW		12.2		11-16.9
Blast cells %		0	1	0-0.05
Promonocytes %		0	1	0-0
Myelocytes %		0	1	0-0

Blood gas analysis and hemoglobins

	Flag	Value	Unit	Reference range
Reticulocyte hemoglobin equivalent, Ret-He		28	pg	28-35
Carbon dioxide content, arterial blood		26	mmol/L	24-32

Biochemistry

	Flag	Value	Unit	Reference range
Glucose		4.2	mmol/L	4.1-5.6
Urea		4.3	mmol/L	2.1-7.1
Potassium, K		4.3	mmol/L	3.5-5
Sodium, Na		140	mmol/L	136-145
Chloride, Cl	H	107	mmol/L	98-106
Calcium, Ca		2.23	mmol/L	2.15-2.5
Calcium corrected, Ca		2.23	mmol/L	2.1-2.6
Phosphate		1.22	mmol/L	0.87-1.45
Creatinine		39	µmol/L	0-133
Uric acid		159	µmol/L	90-360
Bilirubin, Total		12	µmol/L	5.1-17
Bilirubin Direct		4	µmol/L	1.7-5.1
Iron, Fe	L	5	µmol/L	9-30.4
Iron-binding capacity, total		63	µmol/L	40.8-76.7

Protein and amino acid analysis

	Flag	Value	Unit	Reference range
Proteins, total	L	62	g/L	63-79
SPE Albumin		40	g/L	35-50
Transferrin saturation %	L	0.079	1	0.2-0.5
Iron Binding Capacity unsaturated, UIBC	H	58.0	µmol/L	13-56
Ferritin		13	µg/L	10-200
C-Reactive Protein, CRP	H	12	mg/L	0-5

Enzymes

	Flag	Value	Unit	Reference range
Alkaline Phosphatase, AP		1.20	µkat/L	0.71-1.67
Aspartate Aminotransferase,		0.82	µkat/L	0.17-1

Metamyelocytes %	L	0 1	0.13-0.32	AST, GOT				
Band neutrophil %		0 1	0-0.1	Alanine Aminotransferase, ALT, GPT	H	0.55	µkat/L	0.17-0.48
Plasmacytes %		0 1	0-0	Gamma-Glutamyltransferase, GGT		0.16	µkat/L	0-0.65
				Lactate Dehydrogenase, LD	H	17.62	µkat/L	1.7-3.2

Smart Blood Analytics Report

Model used: Hematology (CT181)

Report Date: 2017/05/30

	ICD	Prevalence	Prediction	Information	Description
	D50	14.45%	13.40%	-0.11	**Iron deficiency anaemia**
	D69	9.02%	12.80%	0.50	**Purpura and other haemorrhagic conditions**
	D46	5.56%	10.00%	0.85	**Myelodysplastic syndromes**
	D64	2.65%	8.40%	1.66	**Other anaemias**
	D75	6.64%	8.20%	0.30	**Other diseases of blood and blood-forming organs**
	C92	7.01%	7.80%	0.15	**Myeloid leukaemia**
	C90	8.99%	6.00%	-0.58	**Multiple myeloma and malignant plasma cell neoplasms**
	D53	1.14%	4.60%	2.01	**Other nutritional anaemias**
	C83	2.02%	4.40%	1.12	**Diffuse non-Hodgkin lymphoma**
	D59	0.95%	3.40%	1.84	**Acquired haemolytic anaemia**

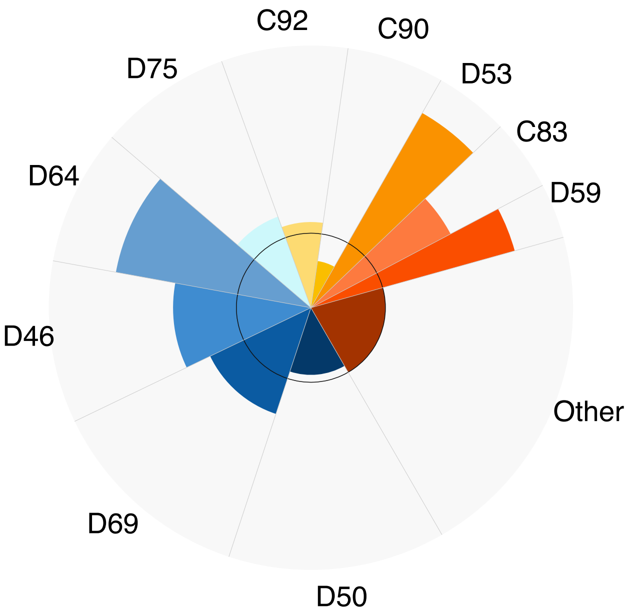
C92
C90
D53
C83
D59
D75
D64
D46
Other
D69
D50

Smart Blood Analytics report #614

#76 primer 9

Female,

Blood Parameters

Lab Test Date: /

Hematology

		Value	Unit	Reference range
Leukocyte count, WBC		4.58	1E9/L	4-10
Neutrophils count		2.56	1E9/L	1.5-7.4
Lymphocyte count		1.74	1E9/L	1-3.4
Monocyte count	L	0.14	1E9/L	0.21-0.92
Eosinophils count		0.14	1E9/L	0.02-0.67
Basophils count		0.01	1E9/L	0-0.13
Immature granulocytes count		0	1E9/L	0-0
Erythroblasts count	H	0.01	1E9/L	0-0
Neutrophils %		0.56	1	0.4-0.7
Lymphocyte %		0.38	1	0.22-0.44
Monocyte %	L	0.03	1	0.04-0.11
Eosinophils %		0.03	1	0-0.08
Basophils %		0	1	0-0.03
Immature Granulocytes %	H	0.013	1	0-0.01
Erythroblast %		0	1	0-0
Hemoglobin	L	65	g/L	120-150
Hematocrit	L	0.177	1	0.36-0.46
Erythrocyte count, RBC	L	1.58	1E12/L	4-5.2
Mean Corpuscular Volume, MCV	H	112	fL	83-101
Mean Corpuscular Hemoglobin, MCH	H	41.1	pg/cell	27-32
Mean Corpuscular Hemoglobin Concentration, MCHC	H	367	g/L	315-345
Erythrocyte Distribution Width, RDW	H	0.159	1	0.116-0.14
Reticulocytes count, urgent automated		32.1	1E9/L	20-100
Reticulocytes %		0.0203	1	0.005-0.025
Hypochromic red cells %, Hypo-He	H	0.029	1	0-0.027
Thrombocytes count, Platelet count	L	59	1E9/L	150-410
Mean Platelet Volume, MPV		9.7	fL	9.2-12.9
Platelet distribution width, PDW		12.7		11-16.9
Blast cells %		0	1	0-0.05
Promonocytes %		0	1	0-0
Myelocytes %		0	1	0-0

Blood gas analysis and hemoglobins

		Value	Unit	Reference range
Reticulocyte hemoglobin equivalent, Ret-He	H	39.1	pg	28-35
Carbon dioxide content, arterial blood		30	mmol/L	24-32

Biochemistry

		Value	Unit	Reference range
Glucose	H	5.9	mmol/L	4.1-5.6
Urea	H	7.8	mmol/L	2.1-7.1
Potassium, K		4.2	mmol/L	3.5-5
Sodium, Na	L	135	mmol/L	136-145
Chloride, Cl		99	mmol/L	98-106
Calcium, Ca		2.16	mmol/L	2.15-2.5
Phosphate		1	mmol/L	0.87-1.45
Creatinine		76	µmol/L	0-133
Uric acid		288	µmol/L	90-360
Bilirubin, Total	H	21	µmol/L	5.1-17
Bilirubin Direct	H	8	µmol/L	1.7-5.1
Iron, Fe	H	37.4	µmol/L	9-30.4
Iron-binding capacity, total		47.8	µmol/L	40.8-76.7

Protein and amino acid analysis

		Value	Unit	Reference range
Proteins, total		69	g/L	63-79
SPE Albumin		37	g/L	35-50
Transferrin saturation %	H	0.782	1	0.2-0.5
Ferritin		118	µg/L	10-200
C-Reactive Protein, CRP		4	mg/L	0-5
Haptoglobin	L	0.07	g/L	0.16-1.99

Enzymes

		Value	Unit	Reference range
Alkaline Phosphatase, AP	L	0.66	µkat/L	0.71-1.67
Aspartate Aminotransferase, AST, GOT	H	1.11	µkat/L	0.17-1
Alanine Aminotransferase, ALT,		0.31	µkat/L	0.17-0.48

Metamyelocytes %	L	0 1	0.13-0.32	
Band neutrophil %		0 1	0-0.1	
Plasmacytes %		0 1	0-0	

GPT			
Gamma-Glutamyltransferase, GGT		0.34 µkat/L	0-0.65
Lactate Dehydrogenase, LD	H	37.12 µkat/L	1.7-3.2

Smart Blood Analytics Report

Model used: Hematology (CT181)

Report Date: 2017/05/30

ICD	Prevalence	Prediction	Information	Description
D53	1.14%	18.20%	3.99	**Other nutritional anaemias**
D46	5.56%	15.40%	1.47	**Myelodysplastic syndromes**
D75	6.64%	13.80%	1.06	**Other diseases of blood and blood-forming organs**
D51	1.09%	11.00%	3.33	**Vitamin B12 deficiency anaemia**
D64	2.65%	9.80%	1.89	**Other anaemias**
D69	9.02%	6.00%	-0.59	**Purpura and other haemorrhagic conditions**
D47	18.50%	4.20%	-2.14	**Other neoplasms of uncertain or unknown behaviour of lymphoid, haematopoietic and related tissue**
D59	0.95%	3.80%	2.00	**Acquired haemolytic anaemia**
C92	7.01%	3.60%	-0.96	**Myeloid leukaemia**
D50	14.45%	3.00%	-2.27	**Iron deficiency anaemia**

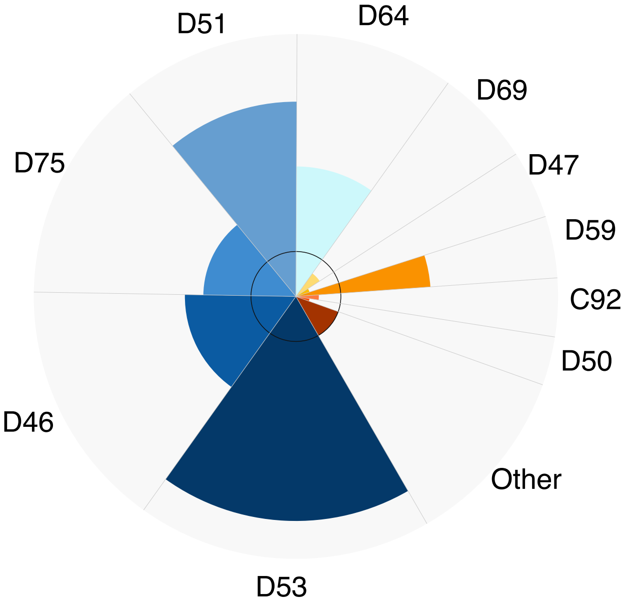
D51
D64
D69
D75
D47
D59
C92
D50
D46
Other
D53

Smart Blood Analytics report #615

#77 primer 10 Female,

Blood Parameters

Lab Test Date: /

Hematology

		Value	Unit	Reference range
Leukocyte count, WBC		9.06	1E9/L	4-10
Neutrophils count		3.90	1E9/L	1.5-7.4
Lymphocyte count	H	3.62	1E9/L	1-3.4
Monocyte count		0.82	1E9/L	0.21-0.92
Eosinophils count	L	0	1E9/L	0.02-0.67
Basophils count		0.07	1E9/L	0-0.13
Immature granulocytes count	H	0.45	1E9/L	0-0
Erythroblasts count	H	0.18	1E9/L	0-0
Neutrophils %	L	0.39	1	0.4-0.7
Lymphocyte %		0.40	1	0.22-0.44
Monocyte %		0.09	1	0.04-0.11
Eosinophils %		0	1	0-0.08
Basophils %		0.03	1	0-0.03
Immature Granulocytes %	H	0.084	1	0-0.01
Erythroblast %	H	0.03	1	0-0
Hemoglobin	L	73	g/L	120-150
Hematocrit	L	0.231	1	0.36-0.46
Erythrocyte count, RBC	L	2.35	1E12/L	4-5.2
Mean Corpuscular Volume, MCV		98.3	fL	83-101
Mean Corpuscular Hemoglobin, MCH		31.1	pg/cell	27-32
Mean Corpuscular Hemoglobin Concentration, MCHC		316	g/L	315-345
Erythrocyte Distribution Width, RDW	H	0.20	1	0.116-0.14
Reticulocytes count, urgent automated		66.7	1E9/L	20-100
Reticulocytes %	H	0.0284	1	0.005-0.025
Hypochromic red cells %, Hypo-He		0.015	1	0-0.027
Thrombocytes count, Platelet count	L	125	1E9/L	150-410
Mean Platelet Volume, MPV	L	8.8	fL	9.2-12.9
Platelet distribution width, PDW	L	8.8		11-16.9
Blast cells %		0	1	0-0.05
Promyelocyte %	L	0	1	0.01-0.08
Myelocytes %	H	0.01	1	0-0

Blood gas analysis and hemoglobins

		Value	Unit	Reference range
Reticulocyte hemoglobin equivalent, Ret-He		28.6	pg	28-35
Carbon dioxide content, arterial blood		27	mmol/L	24-32

Biochemistry

		Value	Unit	Reference range
Glucose	H	7.8	mmol/L	4.1-5.6
Urea		6.5	mmol/L	2.1-7.1
Potassium, K		4.50	mmol/L	3.5-5
Sodium, Na		142	mmol/L	136-145
Chloride, Cl		105	mmol/L	98-106
Calcium, Ca		2.19	mmol/L	2.15-2.5
Creatinine		79	µmol/L	0-133
Uric acid		95	µmol/L	90-360
Bilirubin, Total		7	µmol/L	5.1-17
Bilirubin Direct		3	µmol/L	1.7-5.1
Iron, Fe		11.9	µmol/L	9-30.4

Enzymes

		Value	Unit	Reference range
Acid phosphatase, AP	H	1.61	nkat/L	0-0.9
Alkaline Phosphatase, AP	H	1.83	µkat/L	0.71-1.67
Aspartate Aminotransferase, AST, GOT		0.40	µkat/L	0.17-1
Alanine Aminotransferase, ALT, GPT		0.32	µkat/L	0.17-0.48
Gamma-Glutamyltransferase, GGT		0.26	µkat/L	0-0.65
Lactate Dehydrogenase, LD	H	3.71	µkat/L	1.7-3.2

Protein and amino acid analysis

		Value	Unit	Reference range
Proteins, total	H	91	g/L	63-79
SPE Albumin	L	34	g/L	35-50
Troponin I		0.006	µg/L	0-0.4

		Value	Unit	Reference range
Metamyelocytes %	L	0.04	1	0.13-0.32
Band neutrophil %		0.04	1	0-0.1
Promonocytes %		0	1	0-0
Plasmacytes %		0	1	0-0

Hormones and growth factors

		Value	Unit	Reference range
NT-Pro B-Type Natriuretic Peptide	H	988	µg/L	0-64

		Value	Unit	Reference range
C-Reactive Protein, CRP	H	13	mg/L	0-5
Kappa free		12.30	mg/L	3.3-19.4
Lambda free	H	235	mg/L	5.71-26.3
Kappa/Lambda coefficient	L	0.05		0.26-1.65
Beta-2 microglobulin	H	8.01	mg/L	0-2.7
Haptoglobin		1.5	g/L	0.16-1.99
SPE alpha 1	H	4.1	g/L	2.3-4
SPE alpha 2		7.8	g/L	5-9
SPE beta 1		3.8	g/L	3-6
SPE beta 2	L	2.5	g/L	5-10
SPE gamma	H	39	g/L	7-17
Albumin/Globulin Ratio electrophoresis	L	0.6		1-1

Smart Blood Analytics Report

Model used: Hematology (CT181)

Report Date: 2017/05/30

ICD	Prevalence	Prediction	Information	Description
C90	8.99%	47.80%	2.41	**Multiple myeloma and malignant plasma cell neoplasms**
D47	18.50%	8.20%	-1.17	**Other neoplasms of uncertain or unknown behaviour of lymphoid, haematopoietic and related tissue**
D46	5.56%	6.20%	0.16	**Myelodysplastic syndromes**
D75	6.64%	5.40%	-0.30	**Other diseases of blood and blood-forming organs**
C88	1.00%	5.20%	2.38	**Malignant immunoproliferative diseases**
C92	7.01%	4.80%	-0.55	**Myeloid leukaemia**
C91	8.45%	3.40%	-1.31	**Lymphoid leukaemia**
D64	2.65%	2.80%	0.08	**Other anaemias**
C83	2.02%	2.40%	0.25	**Diffuse non-Hodgkin lymphoma**
D69	9.02%	2.20%	-2.04	**Purpura and other haemorrhagic conditions**

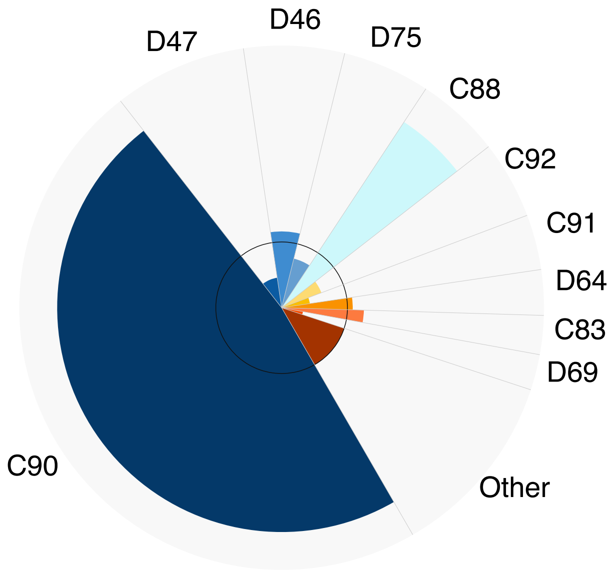
D47
D46
D75
C88
C92
C91
D64
C83
D69
Other
C90

Smart Blood Analytics report #616

#78 primer 11 — Female,

Blood Parameters

Lab Test Date: /

Hematology

	Flag	Value	Unit	Reference range
Leukocyte count, WBC	H	20.74	1E9/L	4-10
Neutrophils count		6.84	1E9/L	1.5-7.4
Lymphocyte count	H	12.86	1E9/L	1-3.4
Monocyte count		0.83	1E9/L	0.21-0.92
Eosinophils count		0.21	1E9/L	0.02-0.67
Immature granulocytes count		0	1E9/L	0-0
Basophils count		0.05	1E9/L	0-0.13
Erythroblasts count		0	1E9/L	0-0
Neutrophils %	L	0.33	1	0.4-0.7
Lymphocyte %	H	0.62	1	0.22-0.44
Monocyte %		0.04	1	0.04-0.11
Eosinophils %		0.01	1	0-0.08
Basophils %		0	1	0-0.03
Immature Granulocytes %		0.003	1	0-0.01
Erythroblast %		0	1	0-0
Hemoglobin		135	g/L	120-150
Hematocrit		0.405	1	0.36-0.46
Erythrocyte count, RBC		4.78	1E12/L	4-5.2
Mean Corpuscular Volume, MCV		84.7	fL	83-101
Mean Corpuscular Hemoglobin, MCH		28.2	pg/cell	27-32
Mean Corpuscular Hemoglobin Concentration, MCHC		333	g/L	315-345
Erythrocyte Distribution Width, RDW		0.131	1	0.116-0.14
Reticulocytes count, urgent automated		78.4	1E9/L	20-100
Reticulocytes %		0.0164	1	0.005-0.025
Hypochromic red cells %, Hypo-He		0.003	1	0-0.027
Thrombocytes count, Platelet count	L	124	1E9/L	150-410
Mean Platelet Volume, MPV		9.6	fL	9.2-12.9
Platelet distribution width, PDW		11.1		11-16.9
Blast cells %		0	1	0-0.05
Promyelocyte %	L	0	1	0.01-0.08
Myelocytes %		0	1	0-0

Blood gas analysis and hemoglobins

	Flag	Value	Unit	Reference range
Reticulocyte hemoglobin equivalent, Ret-He		30	pg	28-35
Carbon dioxide content, arterial blood		26	mmol/L	24-32

Biochemistry

	Flag	Value	Unit	Reference range
Glucose	H	9.1	mmol/L	4.1-5.6
Urea		6.2	mmol/L	2.1-7.1
Potassium, K		4.70	mmol/L	3.5-5
Sodium, Na		144	mmol/L	136-145
Chloride, Cl	H	107	mmol/L	98-106
Calcium, Ca		2.30	mmol/L	2.15-2.5
Phosphate		1.09	mmol/L	0.87-1.45
Creatinine		70	µmol/L	0-133
Uric acid	H	375	µmol/L	90-360
Bilirubin, Total		13	µmol/L	5.1-17
Bilirubin Direct		5	µmol/L	1.7-5.1
Iron, Fe	L	7.3	µmol/L	9-30.4
Iron-binding capacity, total		53.9	µmol/L	40.8-76.7

Protein and amino acid analysis

	Flag	Value	Unit	Reference range
Proteins, total		71	g/L	63-79
SPE Albumin		46	g/L	35-50
Transferrin saturation %	L	0.135	1	0.2-0.5
Ferritin		152	µg/L	10-200
C-Reactive Protein, CRP	H	45	mg/L	0-5

Enzymes

	Flag	Value	Unit	Reference range
Alkaline Phosphatase, AP	H	1.94	µkat/L	0.71-1.67
Aspartate Aminotransferase, AST, GOT		0.39	µkat/L	0.17-1
Alanine Aminotransferase, ALT, GPT		0.28	µkat/L	0.17-0.48

Metamyelocytes %	L	0 1	0.13-0.32	Gamma-Glutamyltransferase, GGT		0.38 µkat/L	0-0.65
Band neutrophil %		0 1	0-0.1	Lactate Dehydrogenase, LD	H	4.74 µkat/L	1.7-3.2
Promonocytes %		0 1	0-0				
Plasmacytes %		0 1	0-0				

Smart Blood Analytics Report

Model used: Hematology (CT181)

Report Date: 2017/05/30

	ICD	Prevalence	Prediction	Information	Description
	C91	8.45%	81.00%	3.26	**Lymphoid leukaemia**
	C83	2.02%	8.60%	2.09	**Diffuse non-Hodgkin lymphoma**
	D69	9.02%	2.20%	-2.04	**Purpura and other haemorrhagic conditions**
	C92	7.01%	1.60%	-2.13	**Myeloid leukaemia**
	D72	0.85%	1.60%	0.91	**Other disorders of white blood cells**
	D75	6.64%	1.20%	-2.47	**Other diseases of blood and blood-forming organs**
	D47	18.50%	1.20%	-3.95	**Other neoplasms of uncertain or unknown behaviour of lymphoid, haematopoietic and related tissue**
	C85	1.09%	0.60%	-0.87	**Other and unspecified types of non-Hodgkin lymphoma**
	D50	14.45%	0.40%	-5.17	**Iron deficiency anaemia**
	D46	5.56%	0.40%	-3.80	**Myelodysplastic syndromes**

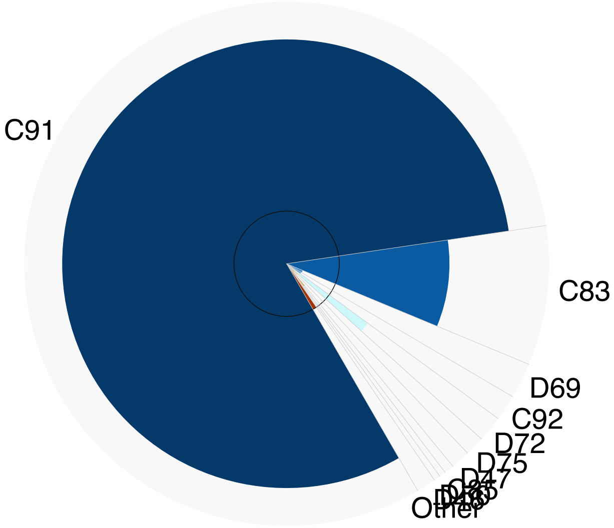
C91
C83
D69
C92
D72
D75
Other

Smart Blood Analytics report #617

#79 primer 12 — Male,

Blood Parameters

Lab Test Date: /

Hematology

	Flag	Value	Unit	Reference range
Leukocyte count, WBC	H	72.60	1E9/L	4-10
Neutrophils count	H	33.58	1E9/L	1.5-7.4
Lymphocyte count	H	5.79	1E9/L	1-3.4
Monocyte count	H	11.53	1E9/L	0.21-0.92
Eosinophils count		0.02	1E9/L	0.02-0.67
Basophils count	H	0.17	1E9/L	0-0.13
Immature granulocytes count	H	21.51	1E9/L	0-0
Erythroblasts count	H	0.03	1E9/L	0-0
Neutrophils %		0.463	1	0.4-0.7
Lymphocyte %	L	0.08	1	0.22-0.44
Monocyte %	H	0.159	1	0.04-0.11
Eosinophils %		0	1	0-0.08
Basophils %		0.002	1	0-0.03
Immature Granulocytes %	H	0.296	1	0-0.01
Erythroblast %		0	1	0-0
Hemoglobin	L	75	g/L	130-170
Hematocrit	L	0.220	1	0.4-0.5
Erythrocyte count, RBC	L	2.26	1E12/L	4.5-5.9
Mean Corpuscular Volume, MCV		97.3	fL	83-101
Mean Corpuscular Hemoglobin, MCH	H	33.2	pg/cell	27-32
Mean Corpuscular Hemoglobin Concentration, MCHC		341	g/L	315-345
Erythrocyte Distribution Width, RDW	H	0.175	1	0.116-0.14
Thrombocytes count, Platelet count	L	32	1E9/L	150-410
Mean Platelet Volume, MPV		11.5	fL	9.4-12.2
Platelet distribution width, PDW		12		11-16.9
Blast cells %		0	1	0-0.05

Biochemistry

	Flag	Value	Unit	Reference range
Glucose		5.3	mmol/L	4.1-5.6
Urea		5.4	mmol/L	2.1-7.1
Potassium, K		3.80	mmol/L	3.5-5
Sodium, Na		141	mmol/L	136-145
Chloride, Cl	H	107	mmol/L	98-106
Creatinine		74	µmol/L	0-133
Bilirubin, Total		8	µmol/L	5.1-17
Bilirubin Direct		4	µmol/L	1.7-5.1

Enzymes

	Flag	Value	Unit	Reference range
Aspartate Aminotransferase, AST, GOT		0.56	µkat/L	0.17-1
Alanine Aminotransferase, ALT, GPT	L	0.17	µkat/L	0.22-0.68
Gamma-Glutamyltransferase, GGT		0.50	µkat/L	0-0.94
Lactate Dehydrogenase, LD	H	20.36	µkat/L	1.7-3.2

Protein and amino acid analysis

	Flag	Value	Unit	Reference range
Troponin I		0.006	µg/L	0-0.4
C-Reactive Protein, CRP	H	15	mg/L	0-5

Hormones and growth factors

	Flag	Value	Unit	Reference range
NT-Pro B-Type Natriuretic Peptide	H	302	µg/L	0-35
Procalcitonin	H	0.16	µg/L	0-0.15

Smart Blood Analytics Report

Model used: Hematology (CT181)

Report Date: 2017/05/30

	ICD	Prevalence	Prediction	Information	Description
	C92	7.01%	42.60%	2.60	**Myeloid leukaemia**
	C90	8.99%	10.40%	0.21	**Multiple myeloma and malignant plasma cell neoplasms**
	C83	2.02%	9.00%	2.16	**Diffuse non-Hodgkin lymphoma**
	D47	18.50%	5.40%	-1.78	**Other neoplasms of uncertain or unknown behaviour of lymphoid, haematopoietic and related tissue**
	C91	8.45%	4.60%	-0.88	**Lymphoid leukaemia**
	D46	5.56%	4.20%	-0.40	**Myelodysplastic syndromes**
	C93	0.45%	4.00%	3.15	**Monocytic leukaemia**
	D69	9.02%	2.40%	-1.91	**Purpura and other haemorrhagic conditions**
	C85	1.09%	2.20%	1.01	**Other and unspecified types of non-Hodgkin lymphoma**
	D75	6.64%	2.20%	-1.59	**Other diseases of blood and blood-forming organs**

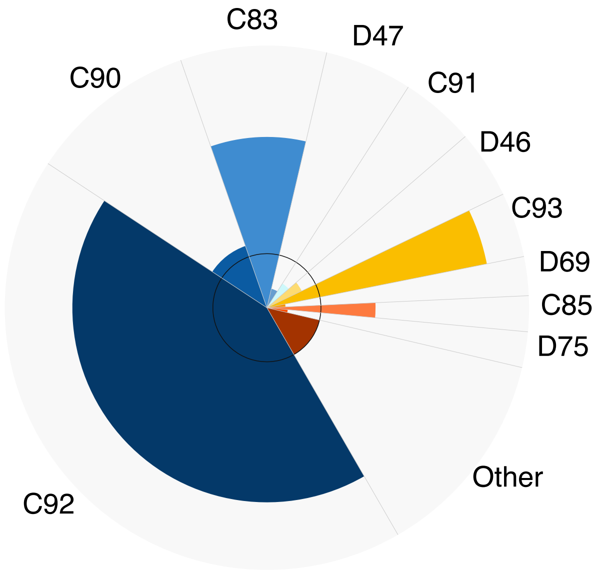
C83
D47
C90
C91
D46
C93
D69
C85
D75
Other
C92

Smart Blood Analytics

Smart Blood Analytics report #618

#80 primer 13 — Female,

Blood Parameters

Lab Test Date: /

Hematology

		Value	Unit	Reference range
Leukocyte count, WBC	H	199.33	1E9/L	4-10
Neutrophils count	H	137.54	1E9/L	1.5-7.4
Lymphocyte count		1.99	1E9/L	1-3.4
Monocyte count	H	1.99	1E9/L	0.21-0.92
Eosinophils count	H	5.98	1E9/L	0.02-0.67
Basophils count	H	5.07	1E9/L	0-0.13
Immature granulocytes count	H	47.84	1E9/L	0-0
Erythroblasts count	H	0.38	1E9/L	0-0
Neutrophils %		0.52	1	0.4-0.7
Lymphocyte %	L	0.01	1	0.22-0.44
Monocyte %	L	0.01	1	0.04-0.11
Eosinophils %		0.03	1	0-0.08
Basophils %		0.01	1	0-0.03
Immature Granulocytes %	H	0.301	1	0-0.01
Erythroblast %	H	0.01	1	0-0
Hemoglobin	L	83	g/L	120-150
Hematocrit	L	0.241	1	0.36-0.46
Erythrocyte count, RBC	L	3.07	1E12/L	4-5.2
Mean Corpuscular Volume, MCV	L	78.5	fL	83-101
Mean Corpuscular Hemoglobin, MCH		27	pg/cell	27-32
Mean Corpuscular Hemoglobin Concentration, MCHC		344	g/L	315-345
Erythrocyte Distribution Width, RDW	H	0.196	1	0.116-0.14
Thrombocytes count, Platelet count		311	1E9/L	150-410
Mean Platelet Volume, MPV		11.8	fL	9.2-12.9
Platelet distribution width, PDW	H	18.1		11-16.9
Blast cells %		0.01	1	0-0.05
Promyelocyte %		0.01	1	0.01-0.08
Myelocytes %	H	0.08	1	0-0
Metamyelocytes %		0.15	1	0.13-0.32
Band neutrophil %	H	0.17	1	0-0.1
Promonocytes %		0	1	0-0
Plasmacytes %		0	1	0-0

Biochemistry

		Value	Unit	Reference range
Glucose		5.5	mmol/L	4.1-5.6
Urea		4.1	mmol/L	2.1-7.1
Potassium, K		4	mmol/L	3.5-5
Sodium, Na		138	mmol/L	136-145
Chloride, Cl		104	mmol/L	98-106
Calcium, Ca	L	1.82	mmol/L	2.15-2.5
Phosphate		1.31	mmol/L	0.87-1.45
Creatinine		36	µmol/L	0-133
Uric acid	L	63	µmol/L	90-360
Bilirubin, Total		9	µmol/L	5.1-17
Bilirubin Direct		5	µmol/L	1.7-5.1
Iron, Fe	L	2.8	µmol/L	9-30.4

Blood gas analysis and hemoglobins

		Value	Unit	Reference range
Carbon dioxide content, arterial blood		26	mmol/L	24-32

Protein and amino acid analysis

		Value	Unit	Reference range
Proteins, total	L	43	g/L	63-79
SPE Albumin	L	24	g/L	35-50
C-Reactive Protein, CRP	H	230	mg/L	0-5

Enzymes

		Value	Unit	Reference range
Alkaline Phosphatase, AP	H	4.55	µkat/L	0.71-1.67
Aspartate Aminotransferase, AST, GOT		0.63	µkat/L	0.17-1
Alanine Aminotransferase, ALT, GPT		0.38	µkat/L	0.17-0.48
Gamma-Glutamyltransferase, GGT	H	1.53	µkat/L	0-0.65
Lactate Dehydrogenase, LD	H	5.96	µkat/L	1.7-3.2

Hormones and growth factors

Hormones and growth factors		Value	Unit	Reference range
NT-Pro B-Type Natriuretic Peptide	H	792	µg/L	0-64

Smart Blood Analytics Report

Model used: Hematology (CT181)   Report Date: 2017/05/30

	ICD	Prevalence	Prediction	Information	Description
	C92	7.01%	42.60%	2.60	**Myeloid leukaemia**
	D47	18.50%	11.00%	-0.75	**Other neoplasms of uncertain or unknown behaviour of lymphoid, haematopoietic and related tissue**
	D50	14.45%	5.20%	-1.47	**Iron deficiency anaemia**
	C91	8.45%	4.20%	-1.01	**Lymphoid leukaemia**
	D75	6.64%	3.60%	-0.88	**Other diseases of blood and blood-forming organs**
	C90	8.99%	3.20%	-1.49	**Multiple myeloma and malignant plasma cell neoplasms**
	D63	0.85%	3.00%	1.82	**Anaemia in chronic diseases classified elsewhere**
	D46	5.56%	2.80%	-0.99	**Myelodysplastic syndromes**
	D64	2.65%	2.40%	-0.14	**Other anaemias**
	C83	2.02%	2.40%	0.25	**Diffuse non-Hodgkin lymphoma**

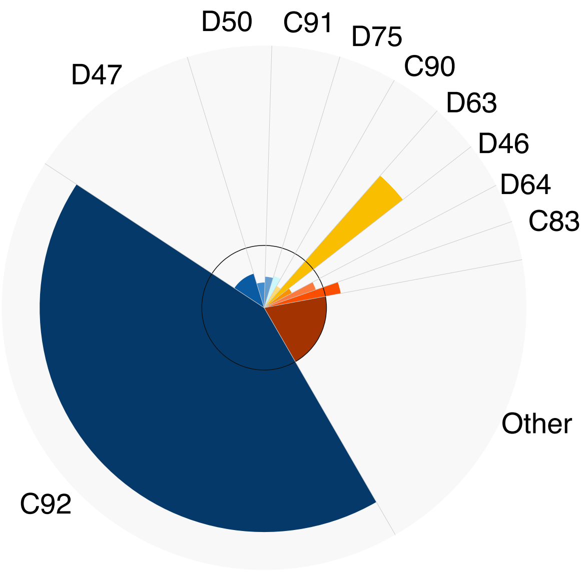
D50
C91
D75
C90
D63
D46
D64
C83
D47
Other
C92

Smart Blood Analytics report #619

#81 primer 14

Female,

Blood Parameters

Lab Test Date: /

Hematology

		Value	Unit	Reference range
Leukocyte count, WBC	H	44.67	1E9/L	4-10
Neutrophils count		2.68	1E9/L	1.5-7.4
Lymphocyte count	H	40.65	1E9/L	1-3.4
Monocyte count		0.89	1E9/L	0.21-0.92
Eosinophils count	L	0	1E9/L	0.02-0.67
Basophils count		0.13	1E9/L	0-0.13
Immature granulocytes count	H	0.45	1E9/L	0-0
Erythroblasts count		0	1E9/L	0-0
Neutrophils %	L	0.06	1	0.4-0.7
Lymphocyte %	H	0.91	1	0.22-0.44
Monocyte %	L	0.02	1	0.04-0.11
Eosinophils %		0	1	0-0.08
Basophils %		0	1	0-0.03
Immature Granulocytes %		0.001	1	0-0.01
Erythroblast %		0	1	0-0
Hemoglobin	L	106	g/L	120-150
Hematocrit	L	0.336	1	0.36-0.46
Erythrocyte count, RBC	L	3.32	1E12/L	4-5.2
Mean Corpuscular Volume, MCV	H	101.2	fL	83-101
Mean Corpuscular Hemoglobin, MCH		31.9	pg/cell	27-32
Mean Corpuscular Hemoglobin Concentration, MCHC		315	g/L	315-345
Erythrocyte Distribution Width, RDW		0.123	1	0.116-0.14
Reticulocytes count, urgent automated		36.5	1E9/L	20-100
Reticulocytes %		0.011	1	0.005-0.025
Hypochromic red cells %, Hypo-He		0.002	1	0-0.027
Thrombocytes count, Platelet count		180	1E9/L	150-410
Mean Platelet Volume, MPV		9.3	fL	9.2-12.9
Platelet distribution width, PDW	L	9.2		11-16.9
Blast cells %		0	1	0-0.05
Promyelocyte %	L	0	1	0.01-0.08
Myelocytes %	H	0.01	1	0-0

Blood gas analysis and hemoglobins

	Value	Unit	Reference range
Reticulocyte hemoglobin equivalent, Ret-He	34.3	pg	28-35
Carbon dioxide content, arterial blood	32	mmol/L	24-32

Biochemistry

		Value	Unit	Reference range
Glucose	H	6.2	mmol/L	4.1-5.6
Urea		5.5	mmol/L	2.1-7.1
Potassium, K		4.30	mmol/L	3.5-5
Sodium, Na		143	mmol/L	136-145
Chloride, Cl		106	mmol/L	98-106
Calcium, Ca		2.31	mmol/L	2.15-2.5
Phosphate		1	mmol/L	0.87-1.45
Creatinine		52	µmol/L	0-133
Uric acid		248	µmol/L	90-360
Bilirubin, Total		17	µmol/L	5.1-17
Bilirubin Direct		5	µmol/L	1.7-5.1
Iron, Fe		14.7	µmol/L	9-30.4
Iron-binding capacity, total		48.8	µmol/L	40.8-76.7

Protein and amino acid analysis

	Value	Unit	Reference range
Proteins, total	69	g/L	63-79
SPE Albumin	46	g/L	35-50
Transferrin saturation %	0.301	1	0.2-0.5
Ferritin	70	µg/L	10-200
C-Reactive Protein, CRP	4	mg/L	0-5

Enzymes

	Value	Unit	Reference range
Alkaline Phosphatase, AP	1.17	µkat/L	0.71-1.67
Aspartate Aminotransferase, AST, GOT	0.20	µkat/L	0.17-1
Alanine Aminotransferase, ALT, GPT	0.20	µkat/L	0.17-0.48
Gamma-Glutamyltransferase,	0.14	µkat/L	0-0.65

Metamyelocytes %	L	0 1	0.13-0.32	GGT			
Band neutrophil %		0 1	0-0.1	Lactate Dehydrogenase, LD	2.20	µkat/L	1.7-3.2
Promonocytes %		0 1	0-0				
Plasmacytes %		0 1	0-0				

Smart Blood Analytics Report

Model used: Hematology (CT181)

Report Date: 2017/05/30

	ICD	Prevalence	Prediction	Information	Description
	C91	8.45%	65.40%	2.95	**Lymphoid leukaemia**
	D72	0.85%	6.60%	2.95	**Other disorders of white blood cells**
	C83	2.02%	6.20%	1.62	**Diffuse non-Hodgkin lymphoma**
	C92	7.01%	5.20%	-0.43	**Myeloid leukaemia**
	D47	18.50%	2.40%	-2.95	**Other neoplasms of uncertain or unknown behaviour of lymphoid, haematopoietic and related tissue**
	C85	1.09%	2.20%	1.01	**Other and unspecified types of non-Hodgkin lymphoma**
	C88	1.00%	1.80%	0.85	**Malignant immunoproliferative diseases**
	C84	0.16%	1.60%	3.34	**Peripheral and cutaneous T-cell lymphomas**
	D46	5.56%	1.60%	-1.80	**Myelodysplastic syndromes**
	D75	6.64%	1.60%	-2.05	**Other diseases of blood and blood-forming organs**

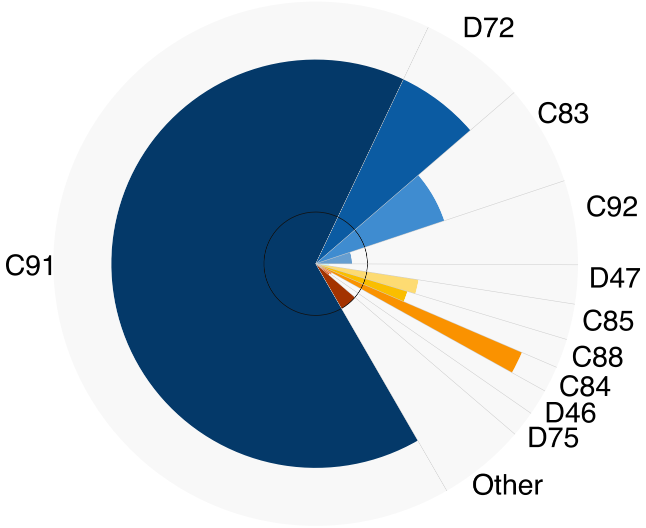
D72
C83
C92
C91
D47
C85
C88
C84
D46
D75
Other

Smart Blood Analytics report #620

#82 primer 15 — Female,

Blood Parameters

Lab Test Date: /

Hematology

		Value	Unit	Reference range
Leukocyte count, WBC		8.22	1E9/L	4-10
Neutrophils count		4.77	1E9/L	1.5-7.4
Lymphocyte count		2.22	1E9/L	1-3.4
Monocyte count		0.41	1E9/L	0.21-0.92
Eosinophils count		0.41	1E9/L	0.02-0.67
Basophils count	H	0.14	1E9/L	0-0.13
Immature granulocytes count		0	1E9/L	0-0
Erythroblasts count		0	1E9/L	0-0
Neutrophils %		0.58	1	0.4-0.7
Lymphocyte %		0.27	1	0.22-0.44
Monocyte %		0.05	1	0.04-0.11
Eosinophils %		0.05	1	0-0.08
Basophils %	H	0.05	1	0-0.03
Immature Granulocytes %		0.002	1	0-0.01
Erythroblast %		0	1	0-0
Hemoglobin		141	g/L	120-150
Hematocrit		0.422	1	0.36-0.46
Erythrocyte count, RBC		4.84	1E12/L	4-5.2
Mean Corpuscular Volume, MCV		87.2	fL	83-101
Mean Corpuscular Hemoglobin, MCH		29.1	pg/cell	27-32
Mean Corpuscular Hemoglobin Concentration, MCHC		334	g/L	315-345
Erythrocyte Distribution Width, RDW		0.129	1	0.116-0.14
Reticulocytes count, urgent automated		63.9	1E9/L	20-100
Reticulocytes %		0.0132	1	0.005-0.025
Hypochromic red cells %, Hypo-He		0.002	1	0-0.027
Thrombocytes count, Platelet count	H	549	1E9/L	150-410
Mean Platelet Volume, MPV		10.3	fL	9.2-12.9
Platelet distribution width, PDW		12		11-16.9
Blast cells %		0	1	0-0.05
Promyelocyte %	L	0	1	0.01-0.08
Myelocytes %		0	1	0-0
Metamyelocytes %	L	0	1	0.13-0.32

Blood gas analysis and hemoglobins

	Value	Unit	Reference range
Reticulocyte hemoglobin equivalent, Ret-He	31.1	pg	28-35
Carbon dioxide content, arterial blood	26	mmol/L	24-32

Biochemistry

		Value	Unit	Reference range
Glucose		4.7	mmol/L	4.1-5.6
Urea		5.3	mmol/L	2.1-7.1
Potassium, K	H	5.10	mmol/L	3.5-5
Sodium, Na		139	mmol/L	136-145
Chloride, Cl		104	mmol/L	98-106
Calcium, Ca		2.37	mmol/L	2.15-2.5
Phosphate		1.14	mmol/L	0.87-1.45
Creatinine		68	µmol/L	0-133
Uric acid		250	µmol/L	90-360
Bilirubin, Total	H	28	µmol/L	5.1-17
Bilirubin Direct	H	9	µmol/L	1.7-5.1
Iron, Fe		20.6	µmol/L	9-30.4
Iron-binding capacity, total		63.3	µmol/L	40.8-76.7

Protein and amino acid analysis

	Value	Unit	Reference range
Proteins, total	70	g/L	63-79
SPE Albumin	45	g/L	35-50
Transferrin saturation %	0.325	1	0.2-0.5
Ferritin	19	µg/L	10-200
C-Reactive Protein, CRP	4	mg/L	0-5

Enzymes

	Value	Unit	Reference range
Alkaline Phosphatase, AP	0.79	µkat/L	0.71-1.67
Aspartate Aminotransferase, AST, GOT	0.31	µkat/L	0.17-1
Alanine Aminotransferase, ALT, GPT	0.24	µkat/L	0.17-0.48

Band neutrophil %	0 1	0-0.1	Gamma-Glutamyltransferase, GGT	0.22	µkat/L	0-0.65
Promonocytes %	0 1	0-0	Lactate Dehydrogenase, LD	2.62	µkat/L	1.7-3.2
Plasmacytes %	0 1	0-0				

Smart Blood Analytics Report

Model used: Hematology (CT181)

Report Date: 2017/05/30

	ICD	Prevalence	Prediction	Information	Description
	D47	18.50%	37.60%	1.02	**Other neoplasms of uncertain or unknown behaviour of lymphoid, haematopoietic and related tissue**
	D75	6.64%	20.80%	1.65	**Other diseases of blood and blood-forming organs**
	D50	14.45%	16.40%	0.18	**Iron deficiency anaemia**
	D68	1.20%	4.40%	1.87	**Other coagulation defects**
	D69	9.02%	4.20%	-1.10	**Purpura and other haemorrhagic conditions**
	D45	2.47%	3.60%	0.54	**Polycythaemia vera**
	D58	0.17%	3.20%	4.23	**Other hereditary haemolytic anaemias**
	D64	2.65%	1.60%	-0.73	**Other anaemias**
	D72	0.85%	1.40%	0.72	**Other disorders of white blood cells**
	D66	0.52%	1.20%	1.20	**Hereditary factor VIII deficiency**

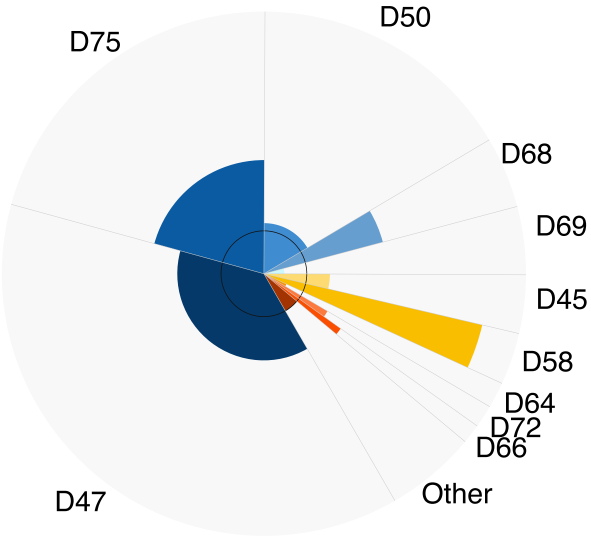
D50
D68
D69
D45
D58
D64
D72
D66
Other
D47
D75

Smart Blood Analytics report #621

#83 primer 16 — Male,

Blood Parameters

Lab Test Date: /

Hematology

		Value	Unit	Reference range
Leukocyte count, WBC		6.92	1E9/L	4-10
Neutrophils count		3.81	1E9/L	1.5-7.4
Lymphocyte count		2.35	1E9/L	1-3.4
Monocyte count		0.42	1E9/L	0.21-0.92
Eosinophils count		0.28	1E9/L	0.02-0.67
Basophils count		0.03	1E9/L	0-0.13
Immature granulocytes count		0	1E9/L	0-0
Erythroblasts count		0	1E9/L	0-0
Neutrophils non-segmented %	H	0.55	1	0.03-0.05
Lymphocyte %		0.34	1	0.22-0.44
Monocyte %		0.06	1	0.04-0.11
Eosinophils %		0.04	1	0-0.08
Basophils %		0.01	1	0-0.03
Immature Granulocytes %		0.003	1	0-0.01
Erythroblast %		0	1	0-0
Hemoglobin		150	g/L	130-170
Hematocrit		0.447	1	0.4-0.5
Erythrocyte count, RBC		5.55	1E12/L	4.5-5.9
Mean Corpuscular Volume, MCV	L	80.5	fL	83-101
Mean Corpuscular Hemoglobin, MCH		27	pg/cell	27-32
Mean Corpuscular Hemoglobin Concentration, MCHC		336	g/L	315-345
Erythrocyte Distribution Width, RDW		0.126	1	0.116-0.14
Reticulocytes count, urgent automated		53.8	1E9/L	20-100
Reticulocytes %		0.0097	1	0.005-0.025
Hypochromic red cells %, Hypo-He		0.004	1	0-0.027
Thrombocytes count, Platelet count	L	35	1E9/L	150-410
Mean Platelet Volume, MPV		11.8	fL	9.4-12.2
Platelet distribution width, PDW	H	20.3		11-16.9
Blast cells %		0	1	0-0.05
Promonocytes %		0	1	0-0
Myelocytes %		0	1	0-0
Metamyelocytes %	L	0	1	0.13-0.32

Blood gas analysis and hemoglobins

		Value	Unit	Reference range
Reticulocyte hemoglobin equivalent, Ret-He		30.6	pg	28-35
Carbon dioxide content, arterial blood		30	mmol/L	24-32

Biochemistry

		Value	Unit	Reference range
Glucose		4.4	mmol/L	4.1-5.6
Urea	H	8	mmol/L	2.1-7.1
Potassium, K		4.60	mmol/L	3.5-5
Sodium, Na		143	mmol/L	136-145
Chloride, Cl		103	mmol/L	98-106
Calcium, Ca		2.49	mmol/L	2.15-2.5
Phosphate		1.31	mmol/L	0.87-1.45
Creatinine		94	µmol/L	0-133
Uric acid		308	µmol/L	150-480
Bilirubin, Total		11	µmol/L	5.1-17
Bilirubin Direct		4	µmol/L	1.7-5.1
Iron, Fe		13	µmol/L	11.6-31.3
Iron-binding capacity, total		53.9	µmol/L	40.8-76.7

Protein and amino acid analysis

		Value	Unit	Reference range
Proteins, total		74	g/L	63-79
SPE Albumin		48	g/L	35-50
Transferrin saturation %		0.241	1	0.2-0.5
Ferritin		58	µg/L	30-300
C-Reactive Protein, CRP		4	mg/L	0-5

Enzymes

		Value	Unit	Reference range
Alkaline Phosphatase, AP	L	0.74	µkat/L	0.9-2.18
Aspartate Aminotransferase, AST, GOT		0.32	µkat/L	0.17-1
Alanine Aminotransferase, ALT, GPT		0.33	µkat/L	0.22-0.68

Band neutrophil %	0 1	0-0.1	Gamma-Glutamyltransferase, GGT	0.25	µkat/L	0-0.94
Plasmacytes %	0 1	0-0	Lactate Dehydrogenase, LD	2.47	µkat/L	1.7-3.2

Smart Blood Analytics Report

Model used: Hematology (CT181)

Report Date: 2017/05/30

	ICD	Prevalence	Prediction	Information	Description
	D69	9.02%	60.40%	2.74	**Purpura and other haemorrhagic conditions**
	D50	14.45%	9.20%	-0.65	**Iron deficiency anaemia**
	D47	18.50%	4.80%	-1.95	**Other neoplasms of uncertain or unknown behaviour of lymphoid, haematopoietic and related tissue**
	D75	6.64%	3.20%	-1.05	**Other diseases of blood and blood-forming organs**
	D68	1.20%	3.20%	1.41	**Other coagulation defects**
	D45	2.47%	2.80%	0.18	**Polycythaemia vera**
	D66	0.52%	2.40%	2.20	**Hereditary factor VIII deficiency**
	C96	0.57%	1.80%	1.65	**Other and unspecified malignant neoplasms of lymphoid, haematopoietic and related tissue**
	D56	0.39%	1.60%	2.04	**Thalassaemia**
	D46	5.56%	1.40%	-1.99	**Myelodysplastic syndromes**

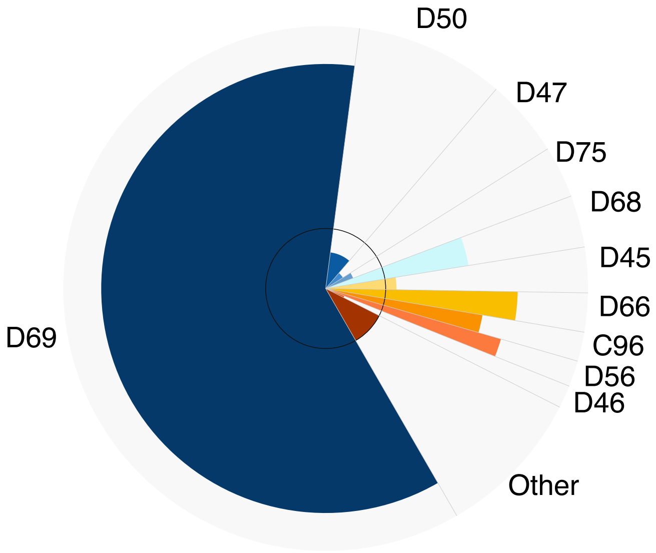
D50
D47
D75
D68
D45
D66
C96
D56
D46
Other
D69

Smart Blood Analytics report #622

#84 primer 17 — Male,

Blood Parameters

Lab Test Date: /

Hematology

	Flag	Value	Unit	Reference range
Leukocyte count, WBC	H	14.85	1E9/L	4-10
Neutrophils count		2.97	1E9/L	1.5-7.4
Lymphocyte count	H	11.29	1E9/L	1-3.4
Monocyte count		0.30	1E9/L	0.21-0.92
Eosinophils count		0.15	1E9/L	0.02-0.67
Basophils count		0.01	1E9/L	0-0.13
Immature granulocytes count		0	1E9/L	0-0
Erythroblasts count	H	0.09	1E9/L	0-0
Neutrophils %	L	0.20	1	0.4-0.7
Lymphocyte %	L	0.15	1	0.22-0.44
Monocyte %	L	0.02	1	0.04-0.11
Eosinophils %		0.01	1	0-0.08
Basophils %		0.01	1	0-0.03
Immature Granulocytes %		0.003	1	0-0.01
Erythroblast %		0	1	0-0
Hemoglobin	L	82	g/L	130-170
Hematocrit	L	0.255	1	0.4-0.5
Erythrocyte count, RBC	L	2.84	1E12/L	4.5-5.9
Mean Corpuscular Volume, MCV		89.8	fL	83-101
Mean Corpuscular Hemoglobin, MCH		28.9	pg/cell	27-32
Mean Corpuscular Hemoglobin Concentration, MCHC		322	g/L	315-345
Erythrocyte Distribution Width, RDW	H	0.28	1	0.116-0.14
Reticulocytes count, urgent automated	L	0.23	1E9/L	20-100
Reticulocytes %		0.0081	1	0.005-0.025
Hypochromic red cells %, Hypo-He	H	0.111	1	0-0.027
Thrombocytes count, Platelet count	L	53	1E9/L	150-410
Blast cells %		0	1	0-0.05
Promyelocyte %	L	0	1	0.01-0.08
Myelocytes %		0	1	0-0
Metamyelocytes %	L	0	1	0.13-0.32
Band neutrophil %		0	1	0-0.1

Blood gas analysis and hemoglobins

	Flag	Value	Unit	Reference range
Reticulocyte hemoglobin equivalent, Ret-He		28.8	pg	28-35
Carbon dioxide content, arterial blood		24	mmol/L	24-32

Biochemistry

	Flag	Value	Unit	Reference range
Glucose		5.4	mmol/L	4.1-5.6
Urea		4.8	mmol/L	2.1-7.1
Potassium, K		4.50	mmol/L	3.5-5
Sodium, Na		142	mmol/L	136-145
Chloride, Cl		106	mmol/L	98-106
Calcium, Ca		2.27	mmol/L	2.15-2.5
Phosphate		1.16	mmol/L	0.87-1.45
Creatinine		87	µmol/L	0-133
Uric acid		336	µmol/L	150-480
Bilirubin, Total		13	µmol/L	5.1-17
Bilirubin Direct		4	µmol/L	1.7-5.1
Iron, Fe		23.9	µmol/L	11.6-31.3
Iron-binding capacity, total		63.6	µmol/L	40.8-76.7

Protein and amino acid analysis

	Flag	Value	Unit	Reference range
Proteins, total		78	g/L	63-79
SPE Albumin		41	g/L	35-50
Transferrin saturation %		0.376	1	0.2-0.5
Ferritin	H	340	µg/L	30-300
C-Reactive Protein, CRP		4	mg/L	0-5

Enzymes

	Flag	Value	Unit	Reference range
Alkaline Phosphatase, AP	H	2.20	µkat/L	0.9-2.18
Aspartate Aminotransferase, AST, GOT		0.69	µkat/L	0.17-1
Alanine Aminotransferase, ALT, GPT		0.68	µkat/L	0.22-0.68

Promonocytes %		0	1	0-0	Gamma-Glutamyltransferase, GGT	H	3.47 µkat/L	0-0.94
Plasmacytes %		0	1	0-0	Lactate Dehydrogenase, LD	H	3.75 µkat/L	1.7-3.2
Hairy cell %	H	0.61	1	0-0				

Smart Blood Analytics Report

Model used: Hematology (CT181)

Report Date: 2017/05/30

	ICD	Prevalence	Prediction	Information	Description
	C91	8.45%	19.40%	1.20	**Lymphoid leukaemia**
	C92	7.01%	18.60%	1.41	**Myeloid leukaemia**
	D46	5.56%	10.60%	0.93	**Myelodysplastic syndromes**
	C83	2.02%	9.40%	2.22	**Diffuse non-Hodgkin lymphoma**
	D47	18.50%	7.80%	-1.25	**Other neoplasms of uncertain or unknown behaviour of lymphoid, haematopoietic and related tissue**
	D75	6.64%	5.20%	-0.35	**Other diseases of blood and blood-forming organs**
	D69	9.02%	4.40%	-1.04	**Purpura and other haemorrhagic conditions**
	C85	1.09%	3.60%	1.72	**Other and unspecified types of non-Hodgkin lymphoma**
	C90	8.99%	2.80%	-1.68	**Multiple myeloma and malignant plasma cell neoplasms**
	D50	14.45%	2.40%	-2.59	**Iron deficiency anaemia**

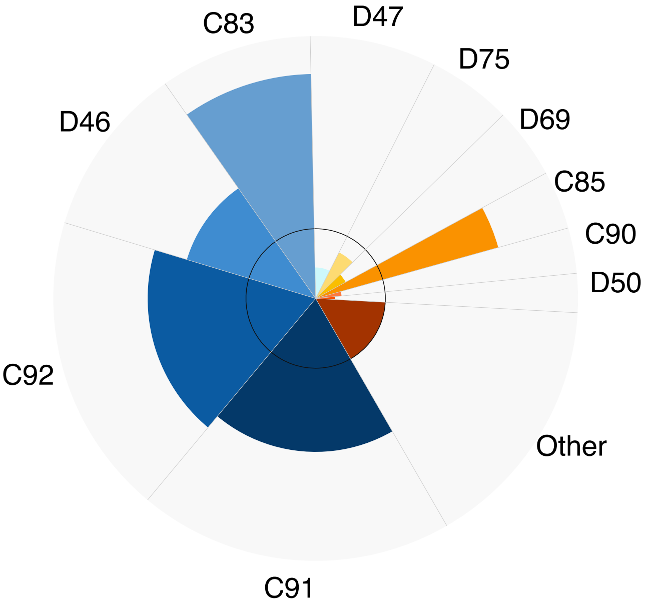
C83
D47
D75
D46
D69
C85
C90
D50
C92
Other
C91

Smart Blood Analytics report #623

#85 primer 18 Female,

Blood Parameters

Lab Test Date: /

Hematology

		Value	Unit	Reference range
Leukocyte count, WBC	H	11.65	1E9/L	4-10
Neutrophils count	H	8.85	1E9/L	1.5-7.4
Lymphocyte count		1.75	1E9/L	1-3.4
Monocyte count	H	1.05	1E9/L	0.21-0.92
Eosinophils count	L	0	1E9/L	0.02-0.67
Basophils count		0.02	1E9/L	0-0.13
Immature granulocytes count		0	1E9/L	0-0
Erythroblasts count	H	0.04	1E9/L	0-0
Neutrophils %	H	0.76	1	0.4-0.7
Lymphocyte %	L	0.15	1	0.22-0.44
Monocyte %		0.09	1	0.04-0.11
Eosinophils %		0	1	0-0.08
Basophils %		0	1	0-0.03
Immature Granulocytes %		0.008	1	0-0.01
Erythroblast %		0	1	0-0
Hemoglobin	L	75	g/L	120-150
Hematocrit	L	0.229	1	0.36-0.46
Erythrocyte count, RBC	L	2.24	1E12/L	4-5.2
Mean Corpuscular Volume, MCV	H	102.2	fL	83-101
Mean Corpuscular Hemoglobin, MCH	H	33.5	pg/cell	27-32
Mean Corpuscular Hemoglobin Concentration, MCHC		328	g/L	315-345
Erythrocyte Distribution Width, RDW	H	0.229	1	0.116-0.14
Reticulocytes count, urgent automated	H	318.8	1E9/L	20-100
Reticulocytes %	H	0.1423	1	0.005-0.025
Hypochromic red cells %, Hypo-He		0.002	1	0-0.027
Thrombocytes count, Platelet count		218	1E9/L	150-410
Mean Platelet Volume, MPV		11.5	fL	9.2-12.9
Platelet distribution width, PDW		14		11-16.9
Blast cells %		0	1	0-0.05
Promyelocyte %	L	0	1	0.01-0.08
Myelocytes %		0	1	0-0

Blood gas analysis and hemoglobins

		Value	Unit	Reference range
Reticulocyte hemoglobin equivalent, Ret-He	H	35.8	pg	28-35

Biochemistry

		Value	Unit	Reference range
Glucose		4.4	mmol/L	4.1-5.6
Urea	H	8.1	mmol/L	2.1-7.1
Potassium, K		4.30	mmol/L	3.5-5
Sodium, Na		141	mmol/L	136-145
Chloride, Cl	H	107	mmol/L	98-106
Calcium, Ca		2.19	mmol/L	2.15-2.5
Creatinine		68	µmol/L	0-133
Uric acid		249	µmol/L	90-360
Bilirubin, Total	H	41	µmol/L	5.1-17
Bilirubin Direct	H	14	µmol/L	1.7-5.1

Protein and amino acid analysis

		Value	Unit	Reference range
Proteins, total		64	g/L	63-79
Troponin I		0.006	µg/L	0-0.4
C-Reactive Protein, CRP	H	8	mg/L	0-5
SPE Albumin		40	g/L	35-50
SPE alpha 1		3.8	g/L	2.3-4
SPE alpha 2	L	4.4	g/L	5-9
SPE beta 1		3.8	g/L	3-6
SPE beta 2	L	2.6	g/L	5-10
SPE gamma		9	g/L	7-17
Albumin/Globulin Ratio electrophoresis	H	1.7		1-1

Enzymes

		Value	Unit	Reference range
Alkaline Phosphatase, AP		1.19	µkat/L	0.71-1.67
Aspartate Aminotransferase, AST, GOT		0.24	µkat/L	0.17-1
Alanine Aminotransferase, ALT,		0.22	µkat/L	0.17-0.48

		Value	Unit	Reference range
Metamyelocytes %	L	0	1	0.13-0.32
Band neutrophil %		0	1	0-0.1
Promonocytes %		0	1	0-0
Plasmacytes %		0	1	0-0

Tumor markers

		Value	Unit	Reference range
Cancer antigen 15-3, CA 15-3		18.6	kU/L	0-30
Carbohydrate Antigen 19-9, CA 19-9		3.0	kU/L	0-37
Carcinoembryonic Antigen, CEA		0.5	µg/L	0-3.4

		Value	Unit	Reference range
GPT				
Gamma-Glutamyltransferase, GGT		0.21	µkat/L	0-0.65
Amylase, Total		1.14	µkat/L	0.46-2.21
Lipase		0.64	µkat/L	0-0.65
Lactate Dehydrogenase, LD	H	4.38	µkat/L	1.7-3.2

Hormones and growth factors

		Value	Unit	Reference range
NT-Pro B-Type Natriuretic Peptide	H	365	µg/L	0-64
Procalcitonin		0.10	µg/L	0-0.15

Smart Blood Analytics Report

Model used: Hematology (CT181)

Report Date: 2017/05/30

	ICD	Prevalence	Prediction	Information	Description
	D59	0.95%	20.00%	4.40	**Acquired haemolytic anaemia**
	D64	2.65%	14.60%	2.46	**Other anaemias**
	C90	8.99%	11.40%	0.34	**Multiple myeloma and malignant plasma cell neoplasms**
	D47	18.50%	7.40%	-1.32	**Other neoplasms of uncertain or unknown behaviour of lymphoid, haematopoietic and related tissue**
	D53	1.14%	6.40%	2.48	**Other nutritional anaemias**
	D46	5.56%	5.60%	0.01	**Myelodysplastic syndromes**
	D50	14.45%	4.80%	-1.59	**Iron deficiency anaemia**
	C91	8.45%	4.40%	-0.94	**Lymphoid leukaemia**
	C92	7.01%	4.00%	-0.81	**Myeloid leukaemia**
	C88	1.00%	4.00%	2.00	**Malignant immunoproliferative diseases**

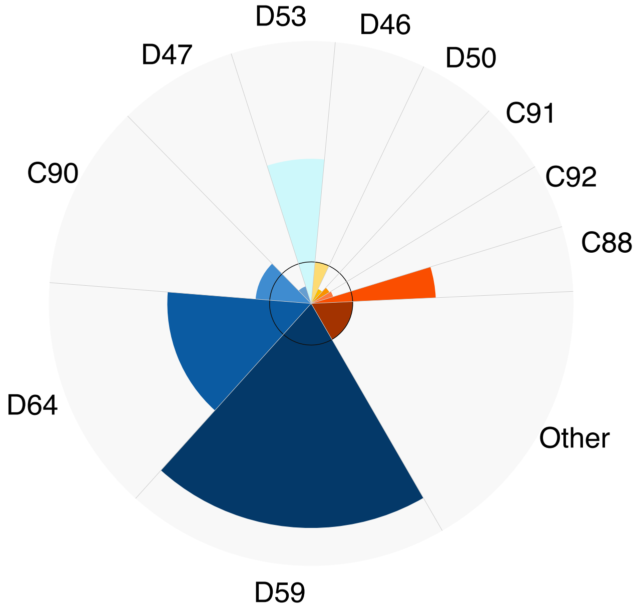
D53
D46
D47
D50
C91
C90
C92
C88
D64
Other
D59

Smart Blood Analytics report #624

#86 primer 19 Male,

Blood Parameters

Lab Test Date: /

Hematology

		Value	Unit	Reference range
Leukocyte count, WBC	H	117.60	1E9/L	4-10
Neutrophils count	H	14.11	1E9/L	1.5-7.4
Lymphocyte count	H	11.76	1E9/L	1-3.4
Monocyte count	H	65.86	1E9/L	0.21-0.92
Eosinophils count	H	1.18	1E9/L	0.02-0.67
Basophils count	H	0.61	1E9/L	0-0.13
Immature granulocytes count	H	9.41	1E9/L	0-0
Erythroblasts count	H	0.50	1E9/L	0-0
Neutrophils %	L	0.12	1	0.4-0.7
Lymphocyte %	L	0.10	1	0.22-0.44
Monocyte %	H	0.21	1	0.04-0.11
Eosinophils %		0.01	1	0-0.08
Basophils %		0.03	1	0-0.03
Immature Granulocytes %	H	0.075	1	0-0.01
Erythroblast %		0	1	0-0
Hemoglobin	L	60	g/L	130-170
Hematocrit	L	0.170	1	0.4-0.5
Erythrocyte count, RBC	L	1.60	1E12/L	4.5-5.9
Mean Corpuscular Volume, MCV	H	106.3	fL	83-101
Mean Corpuscular Hemoglobin, MCH	H	37.5	pg/cell	27-32
Mean Corpuscular Hemoglobin Concentration, MCHC	H	353	g/L	315-345
Erythrocyte Distribution Width, RDW	H	0.17	1	0.116-0.14
Thrombocytes count, Platelet count	L	38	1E9/L	150-410
Mean Platelet Volume, MPV		11.4	fL	9.4-12.2
Platelet distribution width, PDW	L	10.8		11-16.9
Blast cells %	H	0.10	1	0-0.05
Promyelocyte %		0.02	1	0.01-0.08
Myelocytes %	H	0.04	1	0-0
Metamyelocytes %	L	0.02	1	0.13-0.32
Band neutrophil %		0	1	0-0.1
Promonocytes %	H	0.35	1	0-0
Plasmacytes %		0	1	0-0

Biochemistry

		Value	Unit	Reference range
Glucose		4.9	mmol/L	4.1-5.6
Urea	H	7.5	mmol/L	2.1-7.1
Potassium, K		3.8	mmol/L	3.5-5
Sodium, Na		137	mmol/L	136-145
Chloride, Cl		101	mmol/L	98-106
Calcium, Ca	L	2.14	mmol/L	2.15-2.5
Phosphate	L	0.71	mmol/L	0.87-1.45
Creatinine	H	160	µmol/L	0-133
Uric acid	H	605	µmol/L	150-480
Bilirubin, Total		10	µmol/L	5.1-17
Bilirubin Direct		5	µmol/L	1.7-5.1
Iron, Fe		23.5	µmol/L	11.6-31.3
Iron-binding capacity, total		51.9	µmol/L	40.8-76.7

Blood gas analysis and hemoglobins

		Value	Unit	Reference range
Carbon dioxide content, arterial blood	L	23	mmol/L	24-32

Protein and amino acid analysis

		Value	Unit	Reference range
Proteins, total		76	g/L	63-79
SPE Albumin		41	g/L	35-50
Transferrin saturation %		0.453	1	0.2-0.5
Ferritin	H	1514	µg/L	30-300
C-Reactive Protein, CRP	H	55	mg/L	0-5

Enzymes

		Value	Unit	Reference range
Alkaline Phosphatase, AP	H	2.67	µkat/L	0.9-2.18
Aspartate Aminotransferase, AST, GOT	H	1.25	µkat/L	0.17-1
Alanine Aminotransferase, ALT, GPT	H	1.37	µkat/L	0.22-0.68
Gamma-Glutamyltransferase, GGT	H	2.95	µkat/L	0-0.94

Lactate Dehydrogenase, LD H 13.87 µkat/L 1.7-3.2

Smart Blood Analytics Report

Model used: Hematology (CT181)

Report Date: 2017/05/30

	ICD	Prevalence	Prediction	Information	Description
	C92	7.01%	44.80%	2.68	**Myeloid leukaemia**
	C93	0.45%	9.40%	4.38	**Monocytic leukaemia**
	D47	18.50%	7.60%	-1.28	**Other neoplasms of uncertain or unknown behaviour of lymphoid, haematopoietic and related tissue**
	C83	2.02%	7.00%	1.79	**Diffuse non-Hodgkin lymphoma**
	C91	8.45%	6.80%	-0.31	**Lymphoid leukaemia**
	D46	5.56%	6.00%	0.11	**Myelodysplastic syndromes**
	C85	1.09%	2.80%	1.35	**Other and unspecified types of non-Hodgkin lymphoma**
	D75	6.64%	2.60%	-1.35	**Other diseases of blood and blood-forming organs**
	D64	2.65%	2.60%	-0.03	**Other anaemias**
	D53	1.14%	2.00%	0.81	**Other nutritional anaemias**

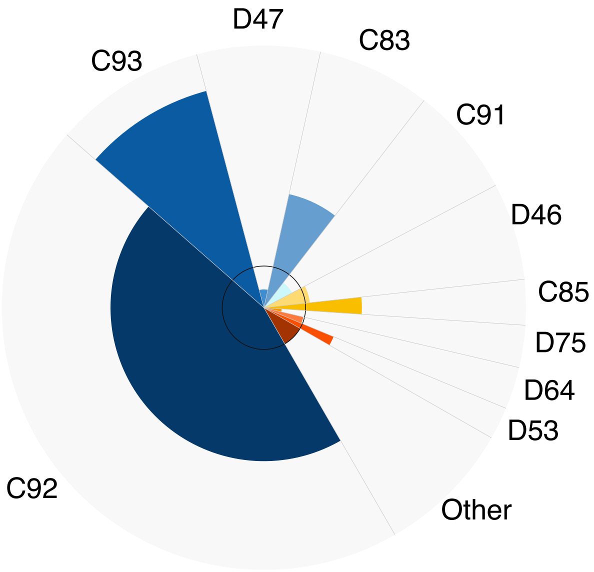
D47
C83
C93
C91
D46
C85
D75
D64
D53
C92
Other

Smart Blood Analytics report #626

#87 primer 20 — Male,

Blood Parameters

Lab Test Date: /

Hematology

		Value	Unit	Reference range
Leukocyte count, WBC		7.11	1E9/L	4-10
Neutrophils count		4.55	1E9/L	1.5-7.4
Lymphocyte count		1.64	1E9/L	1-3.4
Monocyte count		0.64	1E9/L	0.21-0.92
Eosinophils count		0.21	1E9/L	0.02-0.67
Basophils count		0.04	1E9/L	0-0.13
Immature granulocytes count		0	1E9/L	0-0
Erythroblasts count		0	1E9/L	0-0
Neutrophils %		0.64	1	0.4-0.7
Lymphocyte %		0.23	1	0.22-0.44
Monocyte %		0.09	1	0.04-0.11
Eosinophils %		0.03	1	0-0.08
Basophils %		0.01	1	0-0.03
Immature Granulocytes %		0.004	1	0-0.01
Erythroblast %		0	1	0-0
Hemoglobin		139	g/L	130-170
Hematocrit		0.408	1	0.4-0.5
Erythrocyte count, RBC	L	4.48	1E12/L	4.5-5.9
Mean Corpuscular Volume, MCV		91.1	fL	83-101
Mean Corpuscular Hemoglobin, MCH		31	pg/cell	27-32
Mean Corpuscular Hemoglobin Concentration, MCHC		341	g/L	315-345
Erythrocyte Distribution Width, RDW	H	0.149	1	0.116-0.14
Reticulocytes count, urgent automated		50.2	1E9/L	20-100
Reticulocytes %		0.0112	1	0.005-0.025
Hypochromic red cells %, Hypo-He		0.001	1	0-0.027
Thrombocytes count, Platelet count		230	1E9/L	150-410
Mean Platelet Volume, MPV		10.1	fL	9.4-12.2
Platelet distribution width, PDW		11.6		11-16.9
Blast cells %		0	1	0-0.05
Promyelocyte %	L	0	1	0.01-0.08
Myelocytes %		0	1	0-0
Metamyelocytes %	L	0	1	0.13-0.32

Blood gas analysis and hemoglobins

		Value	Unit	Reference range
Reticulocyte hemoglobin equivalent, Ret-He		34	pg	28-35
Carbon dioxide content, arterial blood		29	mmol/L	24-32

Biochemistry

		Value	Unit	Reference range
Glucose		5.1	mmol/L	4.1-5.6
Urea		5.5	mmol/L	2.1-7.1
Potassium, K		4.4	mmol/L	3.5-5
Sodium, Na		140	mmol/L	136-145
Chloride, Cl		100	mmol/L	98-106
Calcium, Ca		2.26	mmol/L	2.15-2.5
Calcium corrected, Ca		2.49	mmol/L	2.1-2.6
Phosphate	H	1.48	mmol/L	0.87-1.45
Creatinine		61	µmol/L	0-133
Uric acid		281	µmol/L	150-480
Bilirubin, Total	L	5	µmol/L	5.1-17
Bilirubin Direct		2	µmol/L	1.7-5.1
Iron, Fe	L	9.7	µmol/L	11.6-31.3
Iron-binding capacity, total		53.5	µmol/L	40.8-76.7

Protein and amino acid analysis

		Value	Unit	Reference range
Proteins, total	L	46	g/L	63-79
SPE Albumin	L	27	g/L	35-50
Transferrin saturation %	L	0.181	1	0.2-0.5
Ferritin		140	µg/L	30-300
C-Reactive Protein, CRP		4	mg/L	0-5
Kappa free		19.1	mg/L	3.3-19.4
Lambda free	H	497	mg/L	5.71-26.3
Kappa/Lambda coefficient	L	0.04		0.26-1.65
Beta-2 microglobulin	H	3.20	mg/L	0-2.7
SPE alpha 1		3.8	g/L	2.3-4
SPE alpha 2		6.9	g/L	5-9

	Value	Unit	Reference range
Band neutrophil %	0	1	0-0.1
Promonocytes %	0	1	0-0
Plasmacytes %	0	1	0-0

	Flag	Value	Unit	Reference range
SPE beta 1		3.5	g/L	3-6
SPE beta 2	L	1.8	g/L	5-10
SPE gamma	L	3	g/L	7-17
Albumin/Globulin Ratio electrophoresis	H	1.4		1-1

Enzymes

	Flag	Value	Unit	Reference range
Alkaline Phosphatase, AP		1.53	µkat/L	0.9-2.18
Aspartate Aminotransferase, AST, GOT		0.42	µkat/L	0.17-1
Alanine Aminotransferase, ALT, GPT		0.36	µkat/L	0.22-0.68
Gamma-Glutamyltransferase, GGT		0.17	µkat/L	0-0.94
Lactate Dehydrogenase, LD	H	3.36	µkat/L	1.7-3.2

Smart Blood Analytics Report

Model used: Hematology (CT181)

Report Date: 2017/05/30

ICD	Prevalence	Prediction	Information	Description
C90	8.99%	36.20%	2.01	**Multiple myeloma and malignant plasma cell neoplasms**
D47	18.50%	29.40%	0.67	**Other neoplasms of uncertain or unknown behaviour of lymphoid, haematopoietic and related tissue**
E85	0.73%	10.20%	3.81	**Amyloidosis**
D50	14.45%	5.60%	-1.37	**Iron deficiency anaemia**
D69	9.02%	3.20%	-1.50	**Purpura and other haemorrhagic conditions**
D75	6.64%	3.20%	-1.05	**Other diseases of blood and blood-forming organs**
D76	0.23%	1.40%	2.60	**Certain diseases involving lymphoreticular tissue and reticulohistiocytic system**
C92	7.01%	1.20%	-2.55	**Myeloid leukaemia**
C83	2.02%	1.00%	-1.01	**Diffuse non-Hodgkin lymphoma**
D64	2.65%	1.00%	-1.41	**Other anaemias**

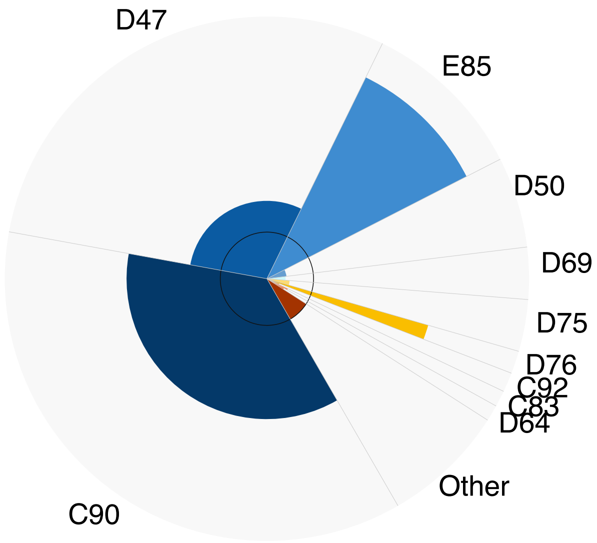
D47
E85
D50
D69
D75
D76
C92
C83
D64
Other
C90

Supplementary Table S3. 43 different haematological categories of diseases that were identified among the 8233 cases analysed

ICD code	Disease
C81	Hodgkin lymphoma
C82	Follicular [nodular] non-Hodgkin lymphoma
C83	Diffuse non-Hodgkin lymphoma
C84	Peripheral and cutaneous T-cell lymphomas
C85	Other and unspecified types of non-Hodgkin lymphoma
C88	Malignant immunoproliferative diseases
C90	Multiple myeloma and malignant plasma cell neoplasms
C91	Lymphoid leukaemia
C92	Myeloid leukaemia
C93	Monocytic leukaemia
C94	Other leukaemias of specified cell type
C95	Leukaemia of unspecified cell type
C96	Other and unspecified malignant neoplasms of lymphoid, haematopoietic and related tissue
D45	Polycythaemia vera
D46	Myelodysplastic syndromes
D47	Other neoplasms of uncertain or unknown behaviour of lymphoid, haematopoietic and related tissue
D50	Iron deficiency anaemia
D51	Vitamin B12 deficiency anaemia
D52	Folate deficiency anaemia
D53	Other nutritional anaemias
D56	Thalassaemia
D58	Other hereditary haemolytic anaemias
D59	Acquired haemolytic anaemia
D60	Acquired pure red cell aplasia [erythroblastopenia]
D61	Other aplastic anaemias
D62	Acute posthaemorrhagic anaemia
D63	Anaemia in chronic diseases classified elsewhere
D64	Other anaemias
D65	Disseminated intravascular coagulation [defibrination syndrome]
D66	Hereditary factor VIII deficiency
D67	Hereditary factor IX deficiency
D68	Other coagulation defects
D69	Purpura and other haemorrhagic conditions
D70	Agranulocytosis
D72	Other disorders of white blood cells
D73	Diseases of spleen
D75	Other diseases of blood and blood-forming organs
D76	Certain diseases involving lymphoreticular tissue and reticulohistiocytic system
D84	Other immunodeficiencies
E75	Disorders of sphingolipid metabolism and other lipid storage disorders
E85	Amyloidosis
I78	Diseases of capillaries
M31	Other necrotising vasculopathies
